# Bacterial Dimethylsulfoniopropionate Biosynthesis in the East China Sea

**DOI:** 10.3390/microorganisms9030657

**Published:** 2021-03-22

**Authors:** Ji Liu, Yunhui Zhang, Jingli Liu, Haohui Zhong, Beth T. Williams, Yanfen Zheng, Andrew R. J. Curson, Chuang Sun, Hao Sun, Delei Song, Brett Wagner Mackenzie, Ana Bermejo Martínez, Jonathan D. Todd, Xiao-Hua Zhang

**Affiliations:** 1 College of Marine Life Sciences, Ocean University of China, 5 Yushan Road, Qingdao 266003, China; jiliuouc@163.com (J.L.); yhzhang2011@163.com (Y.Z.); jingliliu91@163.com (J.L.); zhonghhbio@gmail.com (H.Z.); zhengyf90@126.com (Y.Z.); sunchuang2018@126.com (C.S.); sunhaosy@foxmail.com (H.S.); sdelei@126.com (D.S.); 2 School of Biological Sciences, University of East Anglia, Norwich Research Park, Norwich NR4 7TJ, UK; Beth.Williams@uea.ac.uk (B.T.W.); Andrew.Curson@uea.ac.uk (A.R.J.C.); A.Bermejo-Martinez@uea.ac.uk (A.B.M.); 3 Department of Surgery, School of Medicine, The University of Auckland, Auckland 1142, New Zealand; bc.wagner@auckland.ac.nz; 4 Laboratory for Marine Ecology and Environmental Science, Qingdao National Laboratory for Marine Science and Technology, Qingdao 266071, China

**Keywords:** DMSP biosynthesis, sediment, bacteria

## Abstract

Dimethylsulfoniopropionate (DMSP) is one of Earth’s most abundant organosulfur molecules. Recently, many marine heterotrophic bacteria were shown to produce DMSP, but few studies have combined culture-dependent and independent techniques to study their abundance, distribution, diversity and activity in seawater or sediment environments. Here we investigate bacterial DMSP production potential in East China Sea (ECS) samples. Total DMSP (DMSPt) concentration in ECS seawater was highest in surface waters (SW) where phytoplankton were most abundant, and it decreased with depth to near bottom waters. However, the percentage of DMSPt mainly apportioned to bacteria increased from the surface to the near bottom water. The highest DMSP concentration was detected in ECS oxic surface sediment (OSS) where phytoplankton were not abundant. Bacteria with the genetic potential to produce DMSP and relevant biosynthesis gene transcripts were prominent in all ECS seawater and sediment samples. Their abundance also increased with depth and was highest in the OSS samples. Microbial enrichments for DMSP-producing bacteria from sediment and seawater identified many novel taxonomic groups of DMSP-producing bacteria. Different profiles of DMSP-producing bacteria existed between seawater and sediment samples and there are still novel DMSP-producing bacterial groups to be discovered in these environments. This study shows that heterotrophic bacteria significantly contribute to the marine DMSP pool and that their contribution increases with water depth and is highest in seabed surface sediment where DMSP catabolic potential is lowest. Furthermore, distinct bacterial groups likely produce DMSP in seawater and sediment samples, and many novel producing taxa exist, especially in the sediment.

## 1. Introduction

Dimethylsulfoniopropionate (DMSP) is an abundant organic sulfur compound that acts as a signal molecule [1], marine nutrient [2], and the main precursor of the climate-active gas dimethyl sulfide (DMS) through the action of DMSP lyase enzymes in the marine environment [2,3]. DMS has key roles in global sulfur cycling as the major agent for the transfer of biogenic sulfur from the oceans to land [3,4], as an infochemical [5], and potentially in climate regulation through production of aerosols and cloud condensation nuclei [6,7], though this theory was challenged by Quinn and Bates [8]. Many marine bacteria import DMSP and can degrade it via DMSP cleavage or DMSP demethylation pathways [2,9]. Up to 13% of the bacterial carbon demand in surface waters is supported by DMSP, and the sulfur from DMSP can also be efficiently incorporated into bacterial proteins (mostly into methionine, Met). Moreover, excess MeSH produced by demethylation pathway, if not assimilated, reacts to form dissolved nonvolatile compounds, e.g., DOM–metal–MeSH complexes, which affect trace metal availability and chemistry in seawater [10]. Thus, DMSP is one of the most significant carbon sources and a major sulfur source for marine microbial communities, perhaps influencing metal and DOM cycles in the oceans.

Billions of tons of DMSP are made annually, and it is utilized as an antistress compound by many marine eukaryotes, including phytoplankton, macroalgae, corals and some higher plants, such as *Spartina* and sugar cane [11,12,13,14]. Recently, many marine *Alphaproteobacteria* (predominantly *Rhodobacterales*), some *Gammaproteobacteria* and *Actinobacteria* were also shown to produce DMSP via the Met transamination pathway common to many algae and/or the Met methylation pathway common to DMSP-producing plants [15,16,17,18]. Identification of key DMSP biosynthesis genes in bacteria and algae allowed novel DMSP-producers to be elucidated [17,18]. Initially the *dsyB* gene, encoding a methylthiohydroxybutyrate (MTHB) methyltransferase that catalyzes the committed step in the Met transamination pathway, was identified in >100 sequenced *Alphaproteobacteria* [17]. Functional *dsyB* homologues termed *DSYB* also exist in haptophytes, dinoflagellates, corals and many diatoms, and the gene likely originated in bacteria [14]. A distinct MTHB methyltrasferase isoform enzyme TpMMT was also identified in the diatom *Thalassiosira pseudonana* [19]. Williams et al. [18] discovered the *mmtN* gene that encodes a Met *S*-methyltransferase involved in DMSP synthesis in >50 sequenced *Alphaproteobacteria*, *Gammaproteobacteria* and *Actinobacteria*. Organisms containing either *dsyB*, *DSYB* and/or *mmtN* were shown to produce DMSP, and thus these genes are considered robust reporters for DMSP biosynthesis. Williams et al. [18] found that DMSP standing stock concentrations, DMSP synthesis rates and the abundance of these genes were far higher in coastal sediments than those in overlying seawater samples. Approximately 0.35% and 0.01% of surface seawater bacteria were predicted to contain *dsyB* and *mmtN,* respectively, and thus the genetic potential for DMSP production [14,18]. Furthermore, *dsyB* and/or *mmtN* transcripts were found in all available *Tara Oceans* metatranscriptome datasets apportioned to bacterioplankton [14]. There were ~two-fold more algal *DSYB* transcripts than those for the bacterial *dsyB* gene in North Pacific Ocean coastal seawater samples [14,20]. Together, these results suggest that bacteria may play a significant role in the production of DMSP in surface seawater samples but are likely less important than algae [14], while in coastal surface sediment, bacteria were predicted to be important DMSP producers [18]. However, these conclusions were mainly based on analysis of “omics resources” [21] with limited process data, and from a study that focused on coastal sediment [18]. Until now, there have been few studies on bacterial DMSP production in seawater samples or on differences in DMSP-producing bacteria, their synthesis genes and pathways in seawater compared to those in sediment environments.

Much is known about the catabolism of DMSP via lysis (generating DMS) or the demethylation (potentially generating methanethiol) pathways [3,4]. The demethylation pathway is believed to be the major catabolic pathway in marine environments [22] and the key DMS demethylase *dmdA* gene is generally far more abundant than the seven equivalent DMSP lyase *ddd* genes in marine samples [23]. Indeed, previous qPCR analysis showed the *dmdA* gene to be far more abundant in seawater samples from the East China Sea than *dddP*, which is generally the most environmentally abundant DMSP lyase gene [3,4,24].

**Figure 1:**
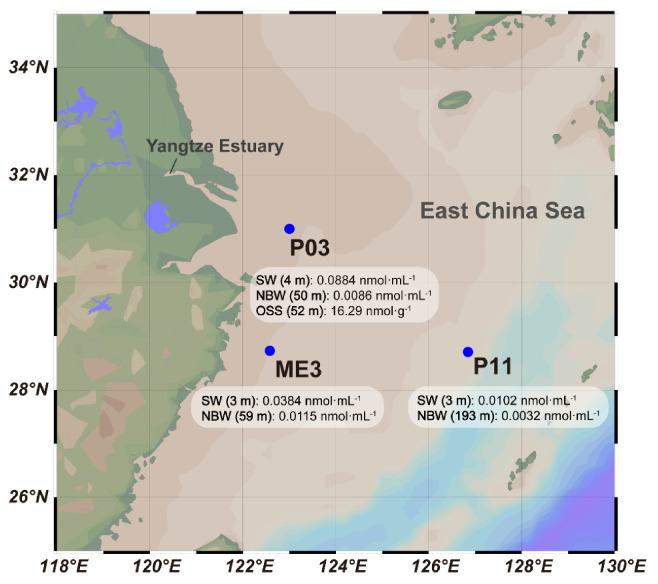
East China Sea sampling site locations and total DMSP concentrations. SW: surface water, NBW: near bottom water, OSS: oxic surface sediment. NBW samples were taken at depths of 59, 52 and 193 m at sites ME3, P03 and P11, respectively.

Here we investigate bacterial DMSP synthesis in surface seawater (SW), near bottom seawater (NBW) and oxic surface sediment (OSS) samples collected from the East China Sea (ECS), where the model *dsyB*-containing strain *Labrenzia aggregata* LZB033 was isolated [17]. DMSP standing stock concentrations with related environmental parameters were measured in three ECS sites (P03, ME3 & P11, Figure 1). The potential contributions of heterotrophic bacteria to DMSP biosynthesis were studied in the different samples. Using culture-dependent and independent methods, the abundance and composition of ECS bacterial DMSP producers in seawater and sediments were predicted, and novel DMSP-producing bacterial taxa were identified. We propose that heterotrophic bacteria make a significant contribution to DMSP production in the ECS, that their contribution increases with depth, with the highest contribution in the OSS.

## 2. Materials and Methods

### 2.1. Sample Collection

Marine samples were obtained on board the R/V “Dong Fang Hong 2” from the ECS at three sites during two cruises from 19 October to 2 November 2015 (sites ME3 & P11) and from 14 July to 1 August 2013 (site P03), respectively (Figure 1). Seawater (SW) 3–4 m deep and near bottom water (NBW), 59 m, 193 m, and 50 m, respectively, from the three sites were collected in Niskin bottles equipped on a Seabird-911 conductivity-temperature-depth (CTD) rosette sampler. Sediment from site P03 was collected with a box corer (top 0–2 cm, in triplicate) as oxic surface sediment (OSS) samples, at a depth of 52 m. For measurement of DMSP and Chlorophyll-*a* (Chl-*a*) concentrations in different size fractions, 1 L seawater from each sample (in triplicate) was prefiltered through 3 μm pore size sterile polycarbonate membranes (Millipore Corporation, Billerica, MA, USA) under controlled pressure (<0.2 bar). The filtrate was then filtered through 0.22 μm pore size sterile polycarbonate membranes (Millipore Corporation, Billerica, MA, USA) under controlled pressure (<0.2 bar). For sediment samples, 0.5 g aliquots were assayed for DMSP in triplicate as described by Williams et al. [18]. Seawater filters and sediment samples for DMSP analysis were stored in a 0.5% (*w*/*v*) sulfuric acid solution at −80 °C until lab analysis.

For culture-dependent and independent work to enrich for DMSP-producing bacteria, 20 mL seawater and 10 g OSS sediment (in triplicate) from site P03 were stored in sterile amber glass vials and kept at 4 °C until lab incubation studies were carried out (see below).

### 2.2. Environmental Parameters

Salinity, temperature and dissolved oxygen data in the seawater samples were recorded by CTD [25]. DMS, DMSP and Chl-*a* concentrations in seawater were measured as previously described by Zhang et al. [26]. For the measurement of DMSPp > 3 μm and DMSPp 0.22–3 μm, corresponding sulfuric acid-preserved membranes were treated with 10 M NaOH and sealed immediately in the vials. After overnight incubation in dark, quantification of DMSP was performed as described by Zhang et al. [26]. DMSP concentrations in OSS samples were measured as previously described by Williams et al. [18]. The detection limit of the method we used to measure DMSP concentration is 0.013 nmol. Concentrations of Chl-*a* in OSS samples were measured as follows: 0.25 g sediment was suspended in 10 mL 90% acetone and ultrasonicated for 15 min at 4 °C in the dark, and then the turbid liquid was incubated at 4 °C for 24 h. After incubation, the turbid liquid was spun at 4000 g for 10 min at 4 °C and then the supernatant was used for Chl-*a* concentration measurement as for SW and NBW samples.

### 2.3. Enrichments for DMSP-Production

To enrich for DMSP-producing bacteria in marine samples, two different incubation experiments were set up in triplicate. For each experiment, 5 mL seawater or 2 g sediment was inoculated into 30 mL modified or normal MBM media (Table S10). Normal MBM medium was 35 PSU, supplemented with 10 mM NH_4_^+^ and a mixed carbon source (10 mM as a final concentration) (Table S10). This was the control experiment to show the background effect of the carbon source addition on the bacterial community change. Modified MBM medium (for the enrichment of DMSP-producing bacteria) [18] had the same mixed carbon source but increased salinity (50 PSU), decreased nitrogen levels (1 mM NH_4_^+^) and 0.5 mM L-Met addition. Experiments were incubated at 90 rpm at 30 °C for two weeks. Sample names were given the suffix T0, T1 and T1C for natural (non-incubated) samples, DMSP-enriched samples and control group samples, respectively. DMSP concentrations in the T1 and T1C samples were monitored by GC as previously described in Williams et al. [18] at time 0, 7 and 14 days.

### 2.4. Bacterial Strain Isolation, Identification and Characterization

T0 seawater and sediment (resuspended in 3% NaCl solution) samples and T1 cultures were serially diluted and spread onto Marine Agar (MA, Becton Dickinson). Plates were incubated at 30 °C for 5–7 days and single colonies were obtained and subsequently purified three times by streaking. All isolates were preliminarily screened for DMSP synthesis capability by growing them in Marine Broth (MB, Becton Dickinson) amended with 0.5 mM Met. Cultures were then assayed for DMSP accumulation by 10 M NaOH alkaline hydrolysis and GC detection of DMS as described by Curson et al. [17]. The 16S rRNA genes of DMSP-producing isolates were amplified using the primer set 27F/1492R [27,28], sequenced (Eurofins Genomics, Munich, Germany) and taxonomically identified against the EzBioCloud 16S rRNA database (https://www.ezbiocloud.net/identify; accessed on 30 May 2016 [29]).

To quantify DMSP production by isolates in rich media, cells were grown in MB for 48 h at 190 rpm, 30 °C. To quantify DMSP production in MBM minimal media (0.5 mM L-Met addition) with two NH_4_^+^ levels (1 mM and 10 mM; to test for the effect of nitrogen availability), bacterial cells were first grown overnight in MB and the culture was washed twice with MBM basal medium (without any nitrogen source, Table S10), then adjusted to OD_600_ of 0.3 and diluted 100-fold into 5 mL of MBM medium and incubated for 48 h at 190 rpm and 30 °C. For both complete and minimal media, 200 µL of the bacterial culture was transferred to 2 mL vials and assayed for DMSP via alkaline lysis and GC analysis for DMS as described by Curson et al. [17]. All experiments described here used three biological replicates. Cellular protein content was estimated by Bradford assays (BioRad, Hemel Hempstead, UK). Estimated intracellular concentrations of DMSP (expressed in mM) were estimated as described by Curson et al. [17].

Isolates were screened by PCR using the primers sets dsyBF/dsyBR and mmtNF/mmtNR (Table S12) to test for the presence of the marker genes *dsyB* and or *mmtN* [17,18]. The PCR reaction included 12.5 µL of 2 × MyFi Mix (Bioline Reagent Ltd., Wellingborough, UK), 0.5 µL of each primer (20 mM), ~50 ng bacterial genomic DNA and nuclease-free water to adjust to a final volume of 25 µL. The reaction conditions were as follows: 95 °C for 5 min; followed by 35 cycles of 95 °C for 20 s, 61 °C (*dsyB*) or 53 °C (*mmtN*) for 30 s, and 72 °C for 30 s; and then a final extension of 72 °C for 10 min. PCR products were visualized by electrophoresis on a 1% agarose gel and purified by High Pure PCR product Purification Kit (Roche Diagnostics GmbH, Germany) before sequencing by Eurofins Genomics. The genomes of representative DMSP-producing bacteria not yielding PCR products for *dsyB* or *mmtN* were sequenced at MicrobesNG (Birmingham, UK). Gene prediction and annotation was carried out using the RAST Server platform (http://rast.nmpdr.org/rast.cgi; accessed on 21 December 2018 [30]). Ratified DsyB and/or MmtN sequences (Table S11) were used in BLASTp searches against these bacterial genomes to identify homologues (E-value ≤ 10^−30^). Each potential DsyB/MmtN homologue was manually checked by BLASTp against the RefSeq database and only those whose top hit was to ratified enzymes were considered as true DsyB/MmtN homologues.

### 2.5. Seawater Incubation Experiments

Representative DMSP producing isolates from this study were grown overnight to stationary phase in normal MBM supplied with mixed carbon source. Bacterial cells were harvested, washed three times and resuspended in filter-sterilized seawater (collected from Zhanqiao Pier, Qingdao, January 2018). The resuspended cultures were adjusted to an OD_600_ of 0.4 and then 100-fold diluted into 20 mL filter-sterilized seawater (t0) in triplicate followed by incubation at 25 °C with shaking at 90 rpm for 21 h (t1) and 43 h (t2). From the t0, t1 and t2 samples, bacterial cells were spun down and the cell-free supernatants were collected. Cell pellets were resuspended in Tris-HCl buffer (50 mM, pH 7.5). DMSP in the cell pellets and cell-free supernatants was hydrolyzed by 10 M NaOH followed by incubation in the dark for 6 h and assayed as described by Williams et al. [18].

### 2.6. DNA and RNA Extraction from Environmental Samples

Total nucleic acids for qPCR, RT-qPCR and 16S rRNA gene amplicon sequencing were extracted from the 0.22–3 µm fraction obtained from 1 L (for qPCR and 16S rRNA gene amplicon sequencing) or 2.5 L (for RT-qPCR) of natural ECS seawater, 0.5 g natural sediments or the cell pellets obtained from 5 mL of incubated sample cultures (harvested at 4000 g at 4 °C for 15 min) as described by Carrión et al. [31]. Total DNA for metagenomic sequencing was extracted from the >0.2 M fraction. Samples were stored at −80 °C and RNA was purified separately and reversely transcribed as described by Williams et al. [18].

### 2.7. Quantitative PCR

To study *dsyB* and *mmtN* relative and absolute abundance and transcription, qPCR and RT-qPCR was performed on a StepOnePlus^TM^ Real-Time PCR system (Applied Biosystems, Waltham, MA, USA) for DNA samples from site P03 T0, T1 and T1C seawater and sediment samples, as well as on cDNA samples from P03 T0 seawater and sediment samples. The reaction compositions, conditions and standard curve making for qPCR and RT-qPCR were carried out according to the method previously described by Williams et al. [18]. All qPCRs were carried out with three technical replicates for each biological replicate.

### 2.8. 16S rRNA Gene Amplicon Sequencing and Statistical Analysis

The V4 hypervariable region of the 16S rRNA gene was amplified from DNA extracted from the 0.22–3 μm fractions of T0, T1 and T1C samples. Amplification, library preparation and sequencing were performed by MR DNA (Shallowater, TX, USA) as described by Carrión et al. [31]. Three biological replicates of each condition were analysed. Raw data were quality-filtered with Trimmomatic [32] and processed on Majorbio I-Sanger Cloud Platform (www.i-sanger.com, accessed on 20 March 2018), obtaining an average of 31,049 sequences per sample with an average length of 298 bp. The resulting sequences were then used to generate operational taxonomic units (OTUs) based on 3% divergence in UPARSE [33]. Chimeras were removed and taxonomic assignment of the final OTUs was carried out using RDP Classifier (http://rdp.cme.msu.edu/, accessed on 20 March 2018) against the Silva database (release 128, http://www.arb-silva.de, accessed on 20 March 2018). Sequences were rarefied to an even depth of 5861 sequences per sample, retaining all samples. The rarefied OTU table was used for all downstream bioinformatic processing.

Rarefaction curves were created to assess sequencing depth, plotting the average number of OTUs (richness) against the number of sequences (Figure S1A). For alpha diversity, The Shannon Index [34] was calculated using the bioinformatic program Mothur (version v.1.30.1) (Figure S1B). For beta diversity, a principal coordinate analysis (PCoA) plot was created with the ‘vegan’ package in R (version 3.6.1) using the Bray-Curtis distance matrix (Figure S1C). Linear discriminate analysis (LDA) effect size (LEfSe; [35]) was used to distinguish the potentially enriched DMSP synthesis-related bacterial lineages with significant difference (*p* < 0.05) between T1 and T1C samples at various taxonomic levels with LDA threshold set ≥3.0.

### 2.9. Metagenomic Sequencing and Analysis

DNA extracted from three biological triplicates of unfiltered P03 T0, T1 and T1C samples were combined respectively in equal proportions based on their DNA concentrations to perform metagenomic sequencing. Library preparations (without any amplification, except for the P03 NBW due to its insufficient DNA quantity) and 2 × 150 bp paired-end shotgun sequencing on a HiSeq 2500 system (Illumina) were performed by MR DNA as described by Carrión et al. [31]. Reads were quality-filtered and trimmed using BayesHammer with default parameters [36], obtaining compressed clean data for each sample ranging in size from ~1.0 to 1.5 Gb. High quality, filtered reads were then assembled using metaSPAdes with default kmers [37]. Gene predictions were performed using Prodigal with default parameters [38]. Basic information about the metagenomic data and assembly quality can be found in Table S9.

Genes were clustered to remove redundant sequences using CD-Hit at the 95% identity and 90% coverage. Genes were annotated using BLASTp with an E-value cutoff of 10^−5^ against NCBI-nr (released August 2016) databases for functional and taxonomic analysis, and only the best hits were retained. Taxonomic assignment was performed in MEGAN software (version 4.6) based on the BLASTp results using the lowest common ancestor (LCA) algorithm. Gene relative abundance was calculated based on the equation below (*a* is gene’s relative abundance, *i* is the code of target gene in metagenome, *b* is gene copy number in metagenome, *j* is the total gene number in metagenome, *k* is the average read length, *x* is unique read hit counts of the specific gene in metagenome, *L* is the gene length):

$$a_{i}=\frac{b_{i}}{\sum b_{j}}=\frac{k\frac{x_{i}}{L_{i}}}{\sum \left(k\frac{x_{j}}{L_{j}}\right)}=\frac{\frac{x_{i}}{L_{i}}}{\sum \left(\frac{x_{j}}{L_{j}}\right)}$$

For retrieval of specific DMSP biosynthesis and degradation genes, profile hidden Markov models (profile HMMs)-based searches of metagenomes for homologues of known DMSP synthesis markers: DsyB and MmtN (bacterial); DSYB and TpMMT (eukaryotic) and DMSP degradation markers: DddD, DddL, DddP, DddQ, DddW, DddY, DddK and DmdA (bacterial) were performed using HMMER (version 3.1b2, http://hmmer.org/, accessed on 9 January 2019). Ratified functional protein sequences of above enzymes shown in Table S10, were used as training sequences to create the HMM profiles. HMM profiles for RecA, RpoB, RplB and RpsC (phylogenetic markers usually used to represent the genome counts) were downloaded from the functional gene pipeline and repository (FunGene, http://fungene.cme.msu.edu/, accessed on 9 January 2021). For the phylogenetic markers, all hits with E-value ≤ 10^−50^ were retrieved. For DMSP-related enzymes, all hits with E-value ≤ 10^−30^ were retrieved and each potential sequence was manually checked by BLASTp against the RefSeq database, and those whose top hit were the ratified DMSP-related enzymes were considered. Then, the hand-filtered sequences for each enzyme were aligned to the corresponding HMM profiles using hmmalign. Approximate maximum likelihood (ML) trees for every enzyme were constructed by FastTree v2.1 and putative peptide sequences which did not cluster most closely to functionally ratified sequences were removed. The final curated homologue sequences were aligned to the training sequences again using the HMM alignment and used to construct the final ML trees using FastTree v2.1. The resulting trees were visualized and annotated using the Interactive Tree of Life (iTOL) version 3.2.4. To estimate the percentage of bacteria containing the target enzymes, the relative abundances of the curated functional enzymes were divided by the average relative abundance of RecA, RpoB, RplB and RpsC retrieved in each metagenomic data set.

Metagenomic binning was performed based on each sample. Reads were mapped to the contigs acquired above with BWA [39], and contigs were grouped into metagenome-assembled genomes (MAGs) based on empirical probabilistic distances of genome abundance and tetranucleotide frequency by MetaBAT 2.12.1 [40]. Completeness and contaminations of MAGs were assessed by CheckM 1.0.7 [41]. MAGs with a completeness ≥80% and contamination ≤10% were used for further analysis. The taxonomy of MAGs was determined by PhyloPhlAn [42] based on protein-coding genes. Identification of DsyB and MmtN homologues in MAGs was performed as described above.

### 2.10. Clone Library Construction and Sequencing

To study the diversity of *dsyB* and *mmtN* amplicons, PCR products of *dsyB* and *mmtN* from P03 SW, NBW and OSS samples were purified and inserted into pMD18-T vectors (TaKaRa Co., Dalian, China), and then transformed into *Escherichia coli* JM109. The insertions in the vector were bidirectionally sequenced. The OTUs of *dsyB* and *mmtN* were determined with nucleotide similarity of 80% and 97%, respectively, by Mothur. Representative sequences of each OTU were translated into protein sequences and used for phylogenetic tree construction as described above.

## 3. Results

### 3.1. Sampling of the ECS and Quantification of DMSP

The potential importance of heterotrophic bacterial DMSP production in the ECS was studied in SW and NBW samples collected from three different sites P03, ME3 and P11, including an OSS sample from P03 (Figure 1, Tables S1 and S2). P03 is close to the Yangtze Estuary and thus has a lower SW salinity (~30 PSU) than the other sites. Site ME3 is also close to land but is influenced less by fresh water input, and thus has higher salinity levels (~33 PSU) and lower nutrient salt concentrations than those of P11, an open water site with less terrestrial impact (Table S1).

Particles within the SW and NBW seawater samples were size fractionated under low pressure (<0.2 bar) in order to allocate DMSP to different sized organisms/particles (>0.7 μm, >3 μm, 0.22–3 μm). As in previous studies [43,44,45,46], GF/F glass fiber filters (nominal retention size of 0.7 µm) were used to determine the concentration of conventional particulate DMSP (DMSPp > 0.7µm). The levels of DMSP captured on 3 μm filters were similar to those present in the >0.7 µm fraction, representing the vast majority of the total DMSP (DMSPt) detected in the seawater samples (62.4–91.6% of DMSPt). GF/F filters (0.7 µm) allow up to 43% of bacteria to pass through [47], and up to 37% of the DMSP in GF/F filtrates is in >0.2 µm particles (thought to be bacteria) [48]. Therefore, we further analyzed DMSPp in the 0.22–3 µm fractions to quantify DMSP mainly in bacterioplankton (DMSPp 0.22–3 µm). Indeed, as indicated by the 16S rRNA amplicon sequencing results, bacteria dominated the 0.22–3 µm fraction in all SW and NBW samples, and very low phytoplankton 16S rRNA sequences were detected in these samples (<0.8% of 16S rRNA sequences) (Table S3).

As expected, DMSPp concentrations in the >0.7 µm and >3 µm fractions were consistently higher in SW samples, which receive the highest light levels compared to NBW (Table S2). This is likely due to higher phytoplankton biomass within these samples, reflected by higher Chl-*a* levels in the three sites (Table S2) and phytoplankton abundance in ME3 and P11 (Table S4). In SW and NBW samples, DMSP apportioned mainly to bacterioplankton (0.22–3 µm) accounted for 4.9–15.8% (equivalent to 0.51–4.35 nM DMSP) of the DMSPt (Table S2). There was a significant trend across all three sites where the contribution of DMSPp 0.22–3 µm to DMSPt increased with the depth from 7.7% ± 2.9% in SW to 13.4% ± 3.0% in NBW (*p* < 0.05, Student’s *t*-test). These data indicated that larger organisms, most likely phytoplankton, contain the bulk of DMSP in ECS seawater, and their biomass and DMSPt levels decrease with depth. Larger bacteria, bacteria clumping together and those associated to phytoplankton will also be present in these samples and may contribute to DMSPt of the larger size fraction. The significance of bacteria as DMSP producers likely increases with depth as their relative contribution to DMSPt levels increases. The highest DMSPt levels (~3 orders of magnitude higher than those in seawater samples per equivalent mass) existed in the P03 OSS samples. These results are consistent with previous findings by Williams et al. [18]. The vast majority of the OSS DMSP (~78.1%, equal to 14.19 nmol DMSP·g^−1^ wet sediment) was in the particulate form. Only 0.05% of the 16S rRNA gene community data from P03 OSS samples corresponded to phytoplankton (Table S1), which is far lower than the ~9% reported in tested saltmarsh surface sediment samples from Williams et al. [18]. This suggests that heterotrophic bacteria likely play a more prominent role in DMSP production in the P03 OSS than in coastal sediments tested in Williams et al. [18].

### 3.2. Enrichment Incubation Experiment for DMSP Producing Bacteria

To identify DMSP-producing bacteria in ECS seawater and sediment, we performed incubation experiments to enrich for DMSP producers in P03 samples. The P03 site was chosen for this experiment because it showed the highest DMSPt and DMSPp concentrations in the 0.22–3 µm fraction. When incubated under combined conditions of high salinity (50 PSU) and reduced nitrogen (1 mM NH_4_^+^) in the presence of Met, DMSP production was significantly enhanced ~12-, 6- and 2-fold (Table S5) in SW, NBW and OSS enrichment samples (T1), respectively, when compared to the control incubations (T1C) after two weeks.

### 3.3. qPCR Analysis of DMSP Biosynthesis Genes

**Figure 2:**
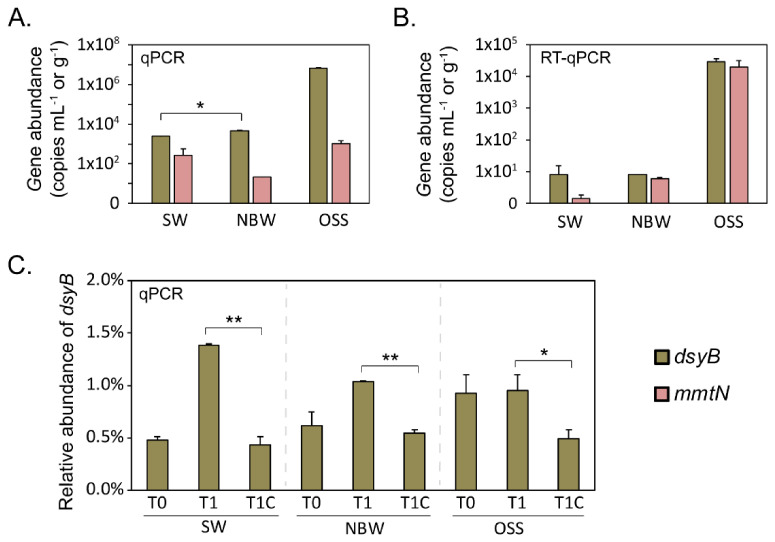
qPCR data on the abundance and transcription of *dsyB* and *mmtN* in P03 samples. (**A**) *dsyB* and *mmtN* gene abundance in P03 SW (surface water), NBW (near bottom water) and OSS (oxic surface sediment) samples. (**B**) *dsyB* and *mmtN* gene transcript abundance in P03 SW, BW and OSS samples. (**C**) Relative abundance of *dsyB* normalized to the abundance of 16s rRNA gene in natural P03 (T0), enriched (T1) and control incubation experiments after 14 days. Data were obtained on two or three independent samples (*n* = 2 or *n* = 3). Error bars represent standard deviation from the mean value. * *p* < 0.05 in Student’s *t*-test. ** *p* < 0.005 in Student’s *t*-test.

The abundance and transcription of the *dsyB* and *mmtN* genes were examined as indicators of bacterial DMSP production in P03 seawater and sediment samples. In P03 seawater samples, the absolute gene abundance of *dsyB* significantly increased from 2.46 × 10^3^ copies/mL in SW to 4.60 × 10^3^ copies/mL in NBW (*p* < 0.05, Student’s *t*-test) (Figure 2A). The relative abundance of *dsyB* (normalized to the 16S rRNA gene abundance) also increased with depth from 0.5% in SW to 0.6% in NBW and to 0.9% in OSS (Figure 2C). *dsyB* was also far more abundant (>3 orders of magnitude more than SW, 6.68 × 10^6^ copies/g) in the OSS samples, where DMSP was most concentrated and phytoplankton abundance was lowest. The abundance of *mmtN* was consistently one to three orders of magnitude lower than *dsyB* in P03 seawater and sediment samples and did not increase with seawater depth. However, like *dsyB*, *mmtN* was most abundant in OSS samples (Figure 2A, Table S6). Importantly, *dsyB* and *mmtN* transcripts were detected in all seawater samples (Figure 2B). Consistent with their gene abundances, *dsyB* and *mmtN* transcripts were also higher (by >3 orders of magnitude) in the OSS samples than in the seawater samples. Interestingly, the levels of *mmtN* and *dsyB* transcripts were more similar to each other than their gene abundance indicated (Figure 2B, Table S6). These data indicate that bacteria are likely producing DMSP throughout the P03 water column and OSS samples, but their contribution to DMSPt, via *dsyB*- and *mmtN*-dependent pathways is highest in the OSS samples.

The relative abundance (normalized to the 16S rRNA gene) of the *dsyB* genes significantly increased in samples from the incubation experiments designed to enrich for DMSP-production (T1) compared to the control incubations (T1C) (Figure 2C).

### 3.4. Isolation and Characterization of DMSP-Producing Bacteria from the ECS

Culture-dependent techniques were used to identify culturable DMSP-producing bacteria in the ECS samples. Up to 6.5% of bacterial isolates in total from ME3, P03 and P11 T0 samples (4.1% in SW, 9.1% in BW and 5.9% in the OSS) produced DMSP when grown in rich MB medium and/or minimal MBM medium with no added methylated sulfur compounds (Table S7). A higher proportion of the isolates (~15.5%, 11.1% and 14.0% for SW, NBW and OSS, respectively) from enriched T1 samples produced DMSP compared to the T0 samples (Table S7). This data, together with the *dsyB* and *mmtN* qPCR work above, is consistent with incubation experiments (under raised salinity and reduced nitrogen levels and in the presence of Met) enriching for DMSP-producing bacteria. Despite the increased ratio of DMSP-producing bacteria isolated, the vast majority of the DMSP-producing isolates from the enriched T1 samples were taxonomically similar to those from the natural samples (T0), and they did not show higher intracellular DMSP levels than those from the natural samples (Table S7).

The DMSP-producing isolates were predominantly in order *Rhodobacterales*, which is reported to account for up to ~30% of bacteria in coastal regions and ~3% of cells in the open ocean surface waters [49,50]. DMSP-producing isolates included *Gammaproteobacteria*, namely *Marinobacter* (*Alteromonadales*), a genus significantly enhanced under DMSP enrichment conditions from surface salt marsh sediment samples [18], and strains of *Halomonas* (*Oceanospirillales*), which have not been reported to synthesize DMSP. Interestingly, a DMSP-producing *Bacillus* was isolated and is the first reported *Firmicute* to produce this osmolyte, though its intracellular DMSP concentration was low compared to most alphaproteobacterial DMSP producers (Table S7). Of the DMSP-producing bacterial isolates, *Labrenzia* and *Marinobacter* strains were the only strains isolated from both seawater and sediment samples, with *Amorphus*, *Acuticoccus*, *Pseudooceanicola*, *Poseidonocella*, *Pelagibaca*, *Thalassospira*, *Oceanicola* and *Halomonas* strains isolated just from seawater, and *Sulfitobacter*, *Stappia* and *Bacillus* strains isolated only in the sediment. This data suggests there may be substantially different bacterial communities producing DMSP within the seawater and sediment environments. The vast majority of these isolates either generated *dsyB* or *mmtN* products using degenerate primers [18] or belong to genera known to contain *dsyB* or *mmtN*, and, thus, likely utilize the Met transamination and/or methylation pathways for DMSP synthesis. Further work is required to establish the DMSP biosynthesis pathway in the gammaproteobacterial *Marinobacter*, *Halomonas* and the *Firmicute Bacillus* isolates, which lack *dsyB* and *mmtN* in their genome sequences obtained in this study. It is possible that they utilize DsyB or MmtN isozymes or novel DMSP biosynthesis pathway(s).

In all tested cases, reduced nitrogen levels in growth media had the effect of increasing the intracellular DMSP concentration of bacterial isolates (Table S7). Considering that the nitrogen level in the natural marine environment is far lower than what was used here (Table S1), it is possible that the natural seawater or sediment environment may result in even higher intracellular DMSP levels. In the low nitrogen and methylated-sulfur-compound-free MBM, bacterial isolates of various taxa produced DMSP at levels ranging from 4.4–391.6 pmol µg protein^−1^ (50.3 pmol µg protein^−1^ in average) with estimated intracellular concentrations of 0.7–61.3 mM (7.9 mM in average). These data together with those of Curson et al. [17] and Williams et al. [18] show that many diverse heterotrophic bacteria can produce DMSP and indicate that some are capable of producing intracellular concentrations higher than those found in many diatoms [51] and comparable to those in some dinoflagellates and haptophytes, generally regarded as high DMSP producers [11,51].

### 3.5. Seawater Incubation Experiment for DMSP Producing Isolates

**Figure 3:**
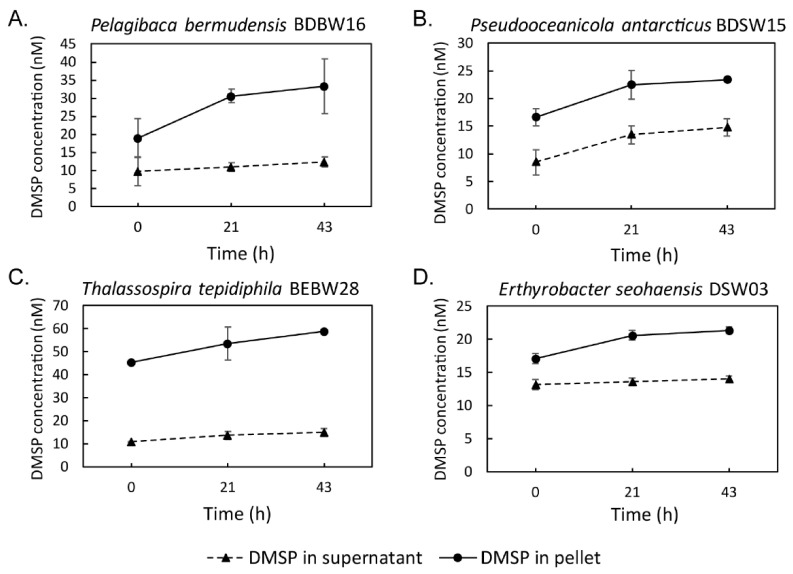
DMSP levels in axenic bacterial cells (pellet) and their cell-free supernatant when incubated in sterilized natural seawater. (**A**) The *dsyB*-containing isolate, *Pelagibaca bermudensis* BDBW16; (**B**) The *dsyB*-containing isolate *Pseudooceanicola antarcticus* BDSW15; (**C**) The *mmtN*-containing isolate *Thalassospira tepidiphila* BEBW28; (**D**) The isolate *Erthyrobacter seohaensis* DSW03 with unknown DMSP biosynthesis gene. Samples were taken at 0 h, after 21 h and 43 h, and DMSP determined by alkaline lysis purge and GC quantification. Error bars display standard deviation from the mean value (n = 3 biologically independent samples). All DMSP-producing isolates show increased intracellular (pellet) and extracellular (supernatant) DMSP levels after 21 and 43 h incubation.

To further demonstrate that DMSP-producing bacterial isolates from this study synthesize DMSP in the natural marine environment, *Rhodobacterales* strains *Pelagibaca bermudensis* BDBW16, *Pseudooceanicola antarcticus* BDSW15, *Rhodospirillales* strain *Thalassospira tepidiphila* BDBW28 and the *Sphingomonadales* strain *Erthyrobacter seohaensis* DSW03 were incubated in filter-sterilized seawater that contained 10.8 ± 1.3 nM DMSP (after 0.22 μm pore size sterile membrane filtration) (Table S1). DMSP was monitored in the bacterial cells and cell-free seawater separately after 21 h and 43 h incubations, respectively. All the isolates showed increased DMSP levels in the cells and the extracellular seawater at both time points (Figure 3). This represented a 4.3–14.4 nM or 25.0–75.5% increase in total DMSP levels due to bacterial DMSP synthesis. These results indicate that not only do the ECS isolates likely produce DMSP in the seawater environment, but they also make a net contribution to the dissolved DMSP pool, thereby providing DMSP for other organisms to import and/or catabolize.

### 3.6. Bacterial Communities and DMSP Producers Revealed by 16S rRNA Gene Amplicon Sequencing

**Figure 4:**
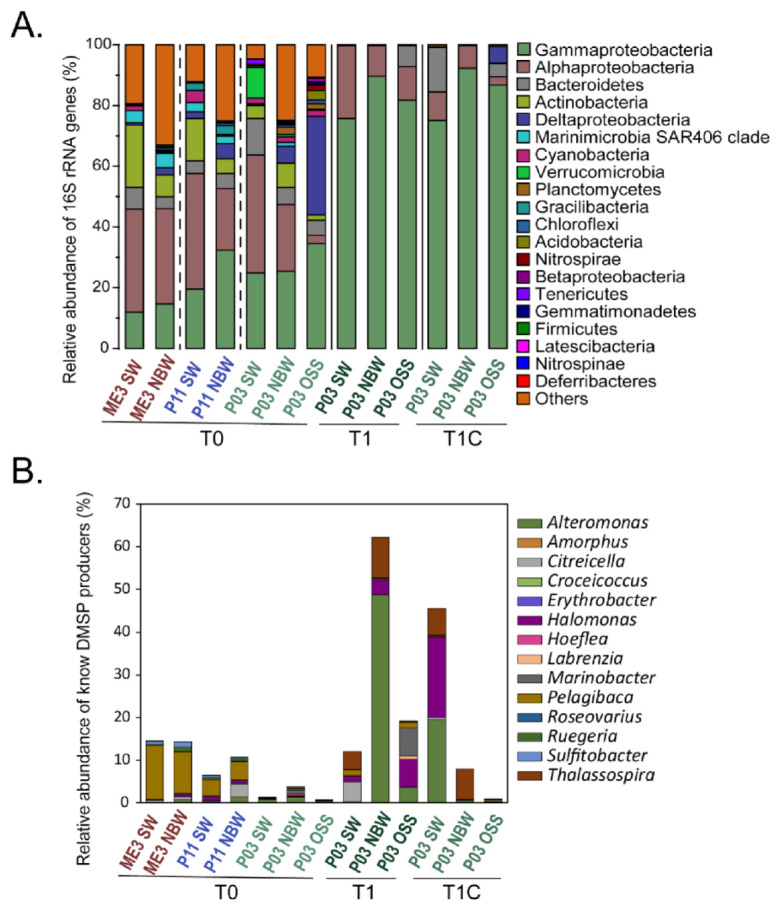
Bacterial community composition and known DMSP producers revealed by 16S rRNA gene amplicon sequencing. (**A**) Relative abundances of taxon-assigned sequences at phylum and class levels from all-natural samples (T0), and from control (T1C) and enriched (T1) incubation experiments of P03 after 14 days. (**B**) Relative abundances of genera with known DMSP-producing species. Data shown in (**A**,**B**) are the averages of three biological replicates. SW: surface water; NBW: near sea water; OSS: oxic surface sediment.

Knowing the identity of many DMSP-producing bacterial genera, we investigated their relative abundances and overall community composition in the ME3, P03 and P11 natural samples by 16S rRNA amplicon sequencing (Figure 4). As expected, the microbial communities within the T0 samples were distinct from those in the incubation experiment T1 and T1C samples (Figure 4A). SW and NBW samples were dominated by *Alphaproteobacteria* and *Gammaproteobacteria*, together constituting 57.3 ± 8.4% of the community. *Actinobacteria* and *Bacteroidetes* were prevalent at varying abundances (0.09–35.0%). In contrast, *Gammaproteobacteria* (~30%) and *Deltaproteobacteria* (~30%) dominated in OSS samples; *Bacteroidetes* were at similar levels to those in the seawater samples, but the abundance of *Alphaproteobacteria* was vastly decreased in OSS samples, to levels constituting < 4% of the community (*p* < 0.05 in Student’s *t*-test).

The microbial community data from the natural samples was analyzed for genera known to contain representative DMSP-producing species, or to contain the *dsyB* and/or *mmtN* genes (from [14,17,18] and this study) (Figure 4B). Site P03, with the highest SW DMSP and high OSS DMSP levels, had by far the lowest proportion of known DMSP-producing genera (1.38%, 3.80% and 0.68% in SW, NBW and OSS respectively). In contrast, higher proportions of DMSP-producing genera were detected in ME3 (14.47% in SW, 14.24% in NBW) and P11 (6.40% in SW, 10.75% in NBW). The most abundant ECS DMSP-producing genus was *Pelagibaca* (9.62–12.5% in ME3, 3.78–4.27% in P11 and 0.01–0.42% in P03); *Sulfitobacter*, *Alteromonas* and *Halomonas* were also abundant; and *Citreicella*, *Marinobacter* and *Ruegeria* were more abundant in NBW than in SW samples. There was an observable increase in the proportion of DMSP-producing genera in the NBW samples compared to SW samples in sites P11 and P03, but not for site ME3 where the levels were similar to SW and NBW samples. The P03 OSS samples had the lowest proportion of known DMSP-producing genera, in contrast with the qPCR and DMSP data, which showed approximately three orders of magnitude higher levels than in any of the tested seawater samples. This anomaly suggests that there are still unidentified DMSP-producing bacteria, particularly in sediment environments, or alternatively that the identified DMSP-producing bacteria are producing higher amounts of DMSP in the OSS compared to seawater.

**Figure 5:**
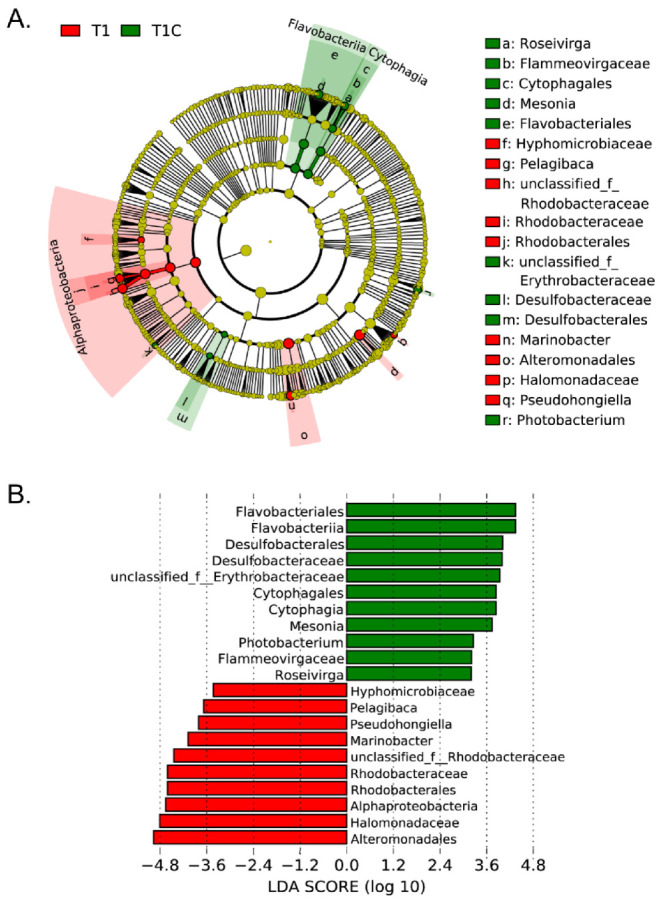
The results of LEfSe analysis of T1 & T1C samples. (**A**) The cladogram shows taxa from phylum to genus levels. Each circle represents a taxon at a different level, and the size of the circle is proportional to the relative abundance. Yellow-colored circles indicate taxa that are not significantly different between groups. Red and green colored circles represent taxa significantly associated with T1 or T1C, respectively. (**B**) The histogram of LDA scores indicating biomarker taxa with significant differences between T1 and T1C groups ranked by effect size.

In the incubated P03 SW, NBW and OSS samples, the T1 and T1C microbial community profiles were very similar to each other at the class level and were distinct from those in the natural P03 samples. This is likely due to the addition of media and/or the presence of mixed carbon sources in the incubated samples, as suggested by the significantly increased levels of *Gammaproteobacteria* (to 83.4 ± 7.2% of the total communities, *p* < 0.05 in Student’s *t*-test), which are known for increasing in abundance in response to this type of enrichment [52]. *Alphaproteobacteria* was the only other class consistently present at >1% abundances in the incubated SW, NBW and OSS samples. A number of significant differences in microbial community composition at genus-level were observed between the control and enriched incubation experiments (*p* < 0.05 in Student’s *t*-test) (Figure 4A, Figure S2). As expected, there were significant increases in the abundance of known DMSP producing genera compared to the natural and the control samples (Figure 4B and Figure 5, Figures S2 and S3). These changes include significant increases in *Alteromonas*, *Thalassospira* and *Halomonas* (to 48.1%, 2.4% and 3.3% of the NBW enriched community, respectively); *Pelagibaca* (to 4.1% and 1.2% of the enriched SW community, respectively); and *Halomonas*, *Marinobacter*, *Altermonas* and *Pelagibaca* (to 6.3%, 6.5%, 3.4% and 1.3% of the enriched OSS community, respectively). Canonical Correspondence Analysis (CCA) analysis further revealed that *Rhodobacteraceae* and *Hyphomonadaceae* of the *Rhodobacterales* and *Halomonadaceae* of the *Oceanospirillales* were positively related to the increased DMSP concentration (Figure S5). It is likely that bacteria from these taxa contribute to the enhanced levels of DMSP detected in the enriched samples.

There were several bacterial genera with significantly increased relative abundances in the P03 enriched samples (to levels >1% of the community) when compared with the control and natural samples, but which were not previously known to have DMSP-producing representatives. These include the following: gammaproteobacterial *Idiomarina*, *Saccharospirillum*, *Teredinibacter*, *Cobetia* and the *Bacteroidetes Haliscomenobacter* and *Balneola* in the enriched OSS community; and gammaproteobacterial *Cobetia* and alphaproteobacterial *Hyphomonas* in the enriched SW community. Some of these genera were also enriched in similar experiments by Williams et al. [18]. It is possible that species within these genera contribute to the enhanced DMSP levels detected in the P03 samples and in the natural samples, but further work is required to test this hypothesis.

### 3.7. Metagenomic Analysis of DMSP Cycling Genes in ECS Samples

**Figure 6:**
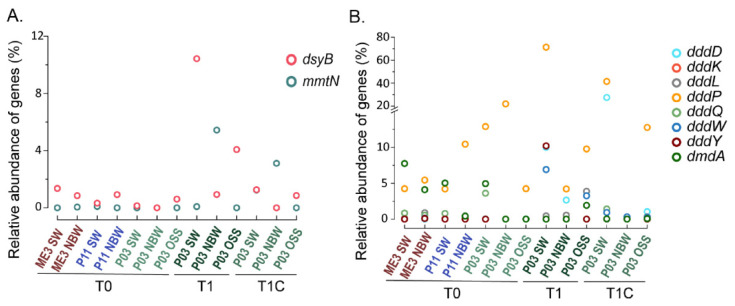
Relative abundance of DMSP cycling genes revealed by metagenomic data of natural samples (T0), and from control (T1C) and enriched (T1) incubation experiments of P03 after 14 days. (**A**) The relative abundance of DMSP biosynthesis genes. (**B**) The relative abundance of DMSP degradation genes. SW: surface water; NBW, near bottom water; OSS, oxic surface sediment.

The relative abundance of DMSP biosynthesis and degradation genes was analyzed in shotgun metagenomics data from the ECS samples. As shown in Figure 6A, the percentage of bacteria predicted to contain *dsyB* ranged from 0.14–1.35% in SW, from below detection −0.92% in NBW, and 0.60% in the P03 OSS samples. The *mmtN* gene, known to be far less abundant than *dsyB* [18], was only detected in the NBW sample of ME3 (0.05%) and SW sample of P11 (0.09%), suggesting that the role of *mmtN* in DMSP biosynthesis in the ECS sample may be less significant than *dsyB*. This data contrasts with that of the RT-qPCR experiments on OSS samples, which showed *mmtN* transcripts to be approaching the levels of *dsyB* (Figure 2, Table S6). This highlights the need for RNA or protein work to complement metagenomic studies and suggests that *dsyB*- and *mmtN*-dependent DMSP production may both be important in OSS samples. For the enriched samples (T1), the total abundance of *dsyB* and/or *mmtN* significantly increased compared to the relevant T0 and T1C samples. *dsyB* was still the dominant DMSP biosynthesis gene in the enriched SW and OSS samples, but *mmtN* was more abundant than *dsyB* in the NBW samples. *dsyB* and *mmtN* gene sequences retrieved from the metagenomic data clustered with known functional *dsyB* or *mmtN* (Figure 6), confirming their identity.

No phytoplankton *DSYB* and *TpMMT* gene sequences were detected in the metagenomic data (produced from unfractionated samples), including the SW where phytoplankton were predicted to be the major contributors to DMSPt levels. This is likely due to the small size of the metagenomes, meaning that the larger eukaryotic genomes may be missed because other recent metagenomic studies with greater sequence depth have detected DSYB and TpMMT in surface water samples [53].

**Figure 7:**
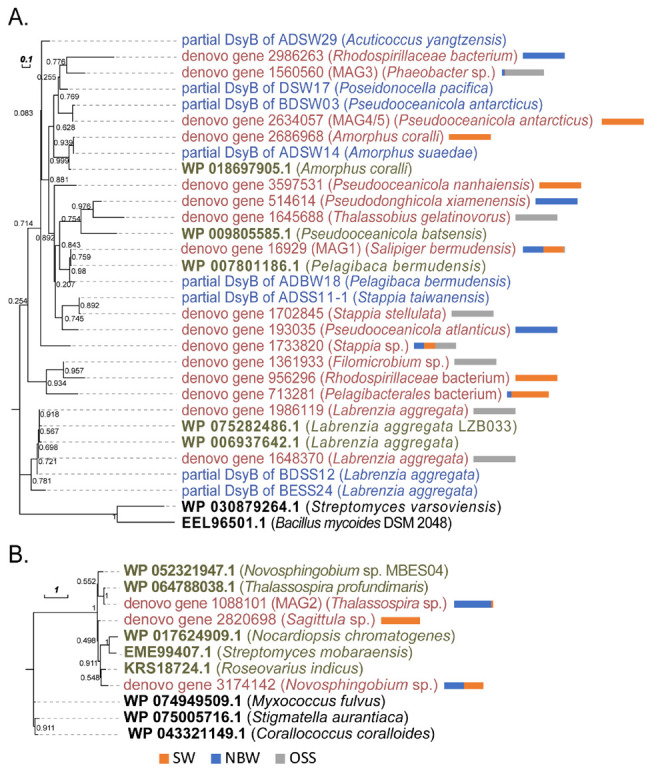
Maximum-likelihood phylogenetic tree of DMSP biosynthesis protein sequences retrieved from metagenomic data. (**A**) DsyB sequences from metagenomic data (red) and degenerate PCR (blue) in this study and functionally ratified DsyB (green). (**B**) MmtN sequences from metagenomic data (red) and functionally ratified MmtN (green). Nonfunctional DsyB and MmtN sequences were indicated in black. Branch lengths are measured in the number of substitutions per site, as indicated on the scale bar. Total abundance of sequences from metagenomic data in SW, NBW and OSS are indicated by the color bars (in percentage).

Fifty high quality MAGs (completeness ≥ 80% and contamination ≤ 10%) were recovered from the metagenomics data, 9 from the SW, NBW and OSS samples in ME3, P11 and P03, and 41 from the P03 enrichment incubation samples. Homologues of DsyB and MmtN were identified in 4 and 1 MAGs, respectively, and all were most similar to known alphaproteobacterial DsyB and MmtN sequences (Table S8; Figure 7). DsyB/MmtN-containing MAGs derived from the seawater samples were identified as *Alphaproteobacteria* of the *Rhodobacteraceae*, *Rhodospirillaceae* or of unknown genera. In the sediment samples, only one alphaproteobacterial MAG containing DsyB was identified. Combined with the isolation work above, it suggests that *Alphaproteobacteria* are clearly important DMSP producers throughout the water column and sediment, but also that there are bacteria with potentially unknown DMSP-biosynthesis genes/pathways that likely contribute to DMSP production in this environment.

For comparison, known DMSP catabolic gene markers, *dddD*, *dddL*, *dddP*, *dddQ*, *dddW*, *dddY* and *dddK* for DMSP lyase enzymes that generate the climate-active gas DMS from DMSP [3,54] and *dmdA* for the DMSP demethylase enzyme [55], were surveyed against the metagenomic datasets. The abundances of DMSP catabolic genes were much higher (5.26–21.9%) than the DMSP biosynthesis genes (Figure 6B). The most abundant DMSP catabolic gene was *dddP* (4.21–21.9%), followed by *dmdA* (0.43–7.73%) and *dddQ* (0.24–3.6%). These DMSP degradation genes, including *dddY, dddW* and *dddD*, were also enriched in P03 T1 samples that showed increased DMSP levels. These data suggest that DMSP catabolism is likely an important process throughout the ECS water column and the OSS. It was surprising that the relative potential for DMSP catabolism was lowest in the P03 OSS samples, where DMSP was most concentrated. This may point towards DMSP having a more important physiological role in organisms producing it in this sediment environment, or there may be additional unidentified DMSP catabolic enzymes. It was also surprising that *dddP* was the dominant catabolic gene identified in the majority of these samples, when in a later cruise of similar ECS sites [25], *dmdA* was found to dominate over *dddP*. It is likely that seasonality and/or the oceanographic parameters at the sampling sites had a key role to play in shaping microbial communities in these samples and thus the abundance of the DMSP cycling genes.

### 3.8. DsyB and mmtN Diversity Revealed by Clone Library Construction

Having established both the presence of DMSP-producing bacteria (through 16S rRNA amplicon sequencing and isolate work) and known DMSP synthesis genes (through metagenomic and qPCR analysis) throughout the water column and in sediment samples, clone libraries were used to study the diversity in these genes. A total of 112 *dsyB* sequences and 47 *mmtN* sequences were analysed from these samples, which clustered into 19 and 14 OTUs, at 80% and 97% cutoffs, respectively (Figure S6). For *dsyB* diversity, sequences from the SW and NBW samples appeared to cluster in OTUs together, which fall in to four distinct groups. One group is most closely related to *Salipiger bermundensis* DsyB and another closely resembles *Labrenzia aggregata* DsyB. The other two groups are not particularly similar to any known DsyB. The clones from OSS mostly appear to cluster in a different, larger group to those from both SW and NBW, suggesting that there may be a completely different community producing DMSP in the OSS versus the seawater samples. The closest DsyB homologue to the OSS sequences was from *Roseovarius indicus* DSM26383 and is quite far removed from the cluster. Thus, there appeared to be greater biodiversity in DsyB sequences in the sediment to the overlying water. This data supports the hypothesis that there are many undiscovered DMSP-producing bacteria in marine environments, particularly in the sediment where bacteria with distinct DsyB sequences are likely important DMSP producers.

In contrast, there is less diversity in the MmtN sequences with the 14 MmtN OTUs clustering into three groups. Most (12/14) align closely to a *Roseovarius indicus* MmtN, with the remaining two clustering to MmtN from *Thalassospira indica*. Furthermore, there does not appear to be much difference between MmtN sequences from SW, NBW and OSS samples.

## 4. Discussion

Most previous research has only considered eukaryotes as significant producers of DMSP, but recently many heterotrophic bacteria have been shown to produce DMSP at intracellular levels comparable to those in phytoplankton, and the key bacterial DMSP synthesis genes *dsyB* and *mmtN* have been identified [17,18]. However, the role of bacterial DMSP production in the marine environment, especially in seawater, is still unclear. Here, we conducted a case study of bacterial DMSP production in ECS surface and near bottom seawater and oxic sediment samples. In the ECS study sites, the abundance of DMSP-producing bacteria and, likely, their contribution to DMSP levels increased with water depth and was highest in the oxic surface sediment where they are likely the major contributors to the DMSP pool. Culture-dependent and independent work identified many novel DMSP-producing bacterial taxa and that there are distinct profiles of DMSP-producing bacteria within the seawater and sediment samples.

Many bacterial species contain DsyB and/or MmtN genes and likely produce DMSP via transamination and/or methylation pathways [17,18]. Similar to Williams et al. [18], our study showed that conditions of reduced nitrogen and raised salinity, which are known to enhance bacterial DMSP production and *dsyB* transcription, combined with the addition of Met, serve to enrich for DMSP-producing bacteria. Here, Met was used instead of MTHB in Williams et al. [18] because Met is the universal starting substrate for all three known DMSP production pathways and MTHB is not a DMSP synthesis intermediate in bacteria with *mmtN*. The majority of DMSP-producing bacteria isolated here were from the *Rhodobacterales*, *Rhodospirillales* and *Rhizobiales* orders, which are known to contain DsyB and/or MmtN genes. However, isolates of gammaproteobacterial *Halomonas* and *Marinobacter* and, interestingly, one Gram-positive *Bacillus*, with no obvious DsyB or MmtN homologues in their genomes, were also shown to produce DMSP at varied levels (Table S7). It is noteworthy that the genomes of these organisms were not complete and *dsyB* and/or *mmtN* homologues could be present in the missing genomic information. Additionally, no *dsyB* or *mmtN* products were amplified from the genomes of the DMSP-producing *Halomonas*, *Marinobacter* and *Bacillus* isolates, but we note that the degenerate primers may not cover the full diversity of these target genes (e.g., *Sulfitobacter dubis* BDSS02). Further primer optimization may give a wider coverage for distinct homologues. It is also possible that the *Halomonas*, *Marinobacter* and *Bacillus* isolates contain isoform MTHB or Met *S*-methyltransferase enzymes or produce DMSP via a different pathway, e.g., the Met decarboxylation pathway identified in the dinoflagellate *Crypthecodinium cohnii* [56]. Without knowing the identity of the functional gene/s for DMSP synthesis within these organisms, it is difficult to estimate their environmental importance in DMSP production. These undetermined pathways could be more widespread among different microbial taxa than previously thought. Indeed, these genera were moderately abundant in ECS samples, accounting for 0.09–1.06% of all bacteria in seawater and 0.15% in P03 sediment.

Interestingly, qPCR and metagenomics (for *dysB*) data show there to be much higher levels of bacteria with *dsyB* and *mmtN* in sediment samples compared to the seawater, yet the sediment samples have by far the lowest abundance of genera known to contain DMSP-producing species. This could be due to those known DMSP-producing genera in the sediment being more active in the sediment than in seawater, as was predicted to be the case for *mmtN* from RT-qPCR data, but it could also suggest that there may be unknown DMSP-producing bacterial genera to be discovered, particularly in the sediment. Indeed, the identification of multiple DsyB OTUs (in clone library work) not closely matching those from known bacteria in the OSS samples supports this hypothesis. It is clear from the low abundance of known DMSP-producing genera in the OSS samples compared to SW samples that there are likely distinct profiles of DMSP-producing bacteria in ECS seawater compared to the sediment. There were also differences in the profiles of cultivable bacteria from seawater and sediment samples, although this is less informative due to the limits of cultivation that generally favor the culturing of alpha- and gamma-proteobacteria. Nevertheless, this work demonstrates that *dsyB* and indeed DMSP production occurs in a wider range of bacteria that reaches beyond the well-known DMSP-producing species in *Rhodobacterales* and *Rhodospirillales*, e.g., in the *Oceanospirillales* and *Firmicutes*.

It is clear that ECS water samples had far higher DMSP concentrations (3.2–10.3 fold) at the surface where phytoplankton are more abundant than at the near bottom where light intensity and phytoplankton abundance is decreased (Tables S2 and S4). Indeed, the majority (62.5–91.6% of DMSPt) of DMSP in both SW and NBW samples was found in the >3 µm fraction which is mainly apportioned to phytoplankton. Flow cytometry data showed that the >3 µm DMSP is most likely produced by diatoms, chlorophyta and dinoflagellates in ME3 and dinoflagellates in P11 SW samples, although the phytoplankton numbers were lower in the later samples, which was also reflected by lower Chl-*a* and DMSP levels. Unfortunately, flow cytometry was not carried out for P03 samples, but the 16S rRNA data from plastid sequences suggests that phytoplankton, particularly diatoms, were most abundant in these samples, which contained the highest seawater DMSP levels (Table S4, Figure S4). These data are consistent with phytoplankton being the major producers of DMSP in photic waters, especially in the surface waters, although we did not detect DSYB or TpMMT gene sequences in the seawater metagenomic data, likely due to insufficient metagenome sequencing depth. Indeed, similar metagenomics studies with greater sequencing depth (eight-fold) on surface seawater did detect DSYB and TpMMT [53]. It is also noteworthy that qPCR data on other ECS samples [57] demonstrated that DMSP-producing bacteria with *dsyB* were also present in the >3 μm seawater samples, indicating that larger bacteria or bacteria tightly associated to phytoplankton may also be contributing to the DMSP levels detected in this fraction.

Although the DMSP component associated to bacterioplankton (0.22–3 µm) was minor compared to that of the phytoplankton in the SW, it was still significant, ranging from 5.0–15.6% of DMSP_t_. We hypothesize that heterotrophic bacteria likely make a more significant contribution to DMSPt in lower light environments such as those in the NBW and OSS samples. The NBW samples had greatly reduced Chl-*a* levels (25-fold and 7-fold in ME3 and P11 respectively; Table S2) compared to surface seawater samples, resulting in the higher ratios of DMSP-producing bacteria (determined from metagenomics) relative to Chl-*a* (3- and 7-fold for ME3 and P11, respectively). Indeed, in NBW samples, the 0.22–3 µm bacterial fractions accounted for much higher percentages (17–58%) of the total DMSP measured than in the SW. Further studies are needed to support this hypothesis, but it is noteworthy that despite the large decreases in phytoplankton and Chl-*a*, total DMSP concentrations only decreased by ~3-fold between surface and bottom seawater samples, indicating that phytoplankton were not the only significant DMSP producers. It is possible that heterotrophic bacteria are importing some DMSP in the seawater samples, but given that both *dsyB* and *mmtB* transcripts were detected in all water samples and that seawater incubations showed bacteria to produce DMSP, it is likely these bacteria are also producing this compound.

Total DMSP concentrations were ~3 orders of magnitude higher in tested OSS samples than in any seawater sample. There were no algal DMSP synthesis genes detected in OSS metagenomes, where Chl-*a* levels and the proportion of algal 16S rRNA plastid sequences in OSS samples were lower than the SW, although still present. In contrast, *dsyB* and *mmtN* gene and transcript abundance were orders of magnitude higher in these samples than in seawater, suggesting that there is indeed bacterial DMSP synthesis actively taking place in the sediment. These data are consistent with findings from Williams et al. [18], and the hypothesis that benthic DMSP-producing bacteria are important contributors to the high DMSPt levels in ECS coastal OSS samples and in marine sediment in general. It is likely that a portion of the OSS DMSP may originate from sinking particles such as dead algae given the depth sampled and from live algae in the photic OSS. However, given the significant *dsyB* and *mmtN* transcript levels observed in the OSS samples, coupled with the high biological turnover rate of DMSP in seawater [58,59] and the abundance of DMSP catabolic genes in the seawater and sediment, DMSP production via bacteria is likely important in these sediment environments.

## 5. Conclusions

This study shows that heterotrophic bacteria likely make a significant input to the marine pools of DMSP and DMS, especially in lower light and sediment environments where they dominate over DMSP-producing phototrophs. Indeed, the bacterial contribution to total DMSP increases with water depth and, critically, is maximal in the oxic surface sediment. It will be of interest to study the significance of bacterial DMSP production across a broader range of marine environments, such as the sea surface microlayer, deep-sea waters and sediment, where contribution of bacterial DMSP production is unknown and may be potentially significant. However, it is important to note that no DMSP synthesis or catabolic rates were determined in this study, and these are essential to gain a truer picture of DMSP cycling in marine samples.

## Data Availability

The partial *dsyB* gene sequences retrieved by degenerate PCR from cultivated isolates are deposited in GenBank under accession numbers MH715961-MH715968. Genome sequencing data for the representative strains, 16S rRNA amplicon sequencing and metagenomic data are publicly available at DDBJ/ENA/GenBank Whole Genome Shotgun project and NCBI SRA (BioProject PRJNA484519). MAGs are deposited under accession numbers JAAEYP000000000-JAAFAM000000000. DsyB and MmtN sequences derived from metagenomic data are deposited under accession numbers MN970667-MN970682 and MN954779-MN954781, respectively.

## Supplementary Materials

The following are available online at https://www.mdpi.com/2076-2607/9/3/657/s1, Table S1: Environmental parameters of ME3, P11 and P03 seawater and oxic surface sediment samples; Table S2: Chl-a and DMSP-related factors of ME3, P11 and P03 seawater and oxic surface sediment samples; Table S3: Proportion of phytoplankton plastid sequences in total 16S rRNA gene data from amplicon sequencing analysis; Table S4: The phytoplankton community structures of ME3 & P11 derived from flow cytometry; Table S5: DMSP concentrations before and after enrichment incubation; Table S6: qPCR and RT-qPCR analysis of dsyB and mmtN in P03 seawater and sediment samples; Table S7: Characteristics of DMSP-producing bacterial isolates; Table S8: MAGs recovered from the metagenomic data; Table S9: Basic information of metagenomic data; Table S10: Composition of normal or modified MBM medium; Table S11: Reference protein sequences of the functionally ratified DMSP degradation and biosynthesis enzymes; Table S12: Oli-gonucleotide primers used in this study; Figure S1: Alpha- and beta diversity analyses from 16S rRNA amplicon sequencing data; Figure S2: Heatmap depicting the 50 most abundant genera from the 16S rRNA gene amplicon sequencing data from natural ECS samples (T0), the enriched (T1) and control incubation experiments after 14 days; Figure S3: Relative abundance for each significant taxon in the LEfSe analysis of T1 & T1C samples; Figure S4: Composition of eukaryotic plastid 16S rRNA genes in P03 SW and NBW samples; Figure S5: Canonical correspondence analysis (CCA) on bacterial communities at family level in samples of incubation experiments; Figure S6: Amino acid tree of representative DsyB and MmtN OTU sequences from clone libraries.
