# Supplementary material for: Bacterial Dimethylsulfoniopropionate Biosynthesis in the East China Sea
Source: Microorganisms. 2021 Mar 22;9(3):657. doi: 10.3390/microorganisms9030657 (PMC8004995; doi:10.3390/microorganisms9030657)
Supplement: Supplementary file 1 [file microorganisms-09-00657-s001.pdf]

Table S1. Environmental parameters of ME3, P11 and P03 seawater and oxic surface sediment samples

Sample	Sampling time	Location	Depth (m)	Salinity (PSU)	DO ($mg \cdot L^{-1}$)	NO_3^- ($\mu mol \cdot L^{-1}$)	NO_2^- ($\mu mol \cdot L^{-1}$)	NH_3 ($\mu mol \cdot L^{-1}$)	PO_4^{3-} ($\mu mol \cdot L^{-1}$)
ME3 SW	Jul. 2013	28.7 °N,	3	33.63	6.70	1.18	0.42	0.39	0.42
ME3 NBW		122.6 °E	59	34.41	5.03	7.28	0.49	0.39	0.93
P11 SW	Jul. 2013	28.7 °N,	3	33.61	6.09	0.29	0.16	0.54	0.16
P11 NBW		126.9 °E	193	34.39	4.75	18.29	0.18	0.23	1.54
P03 SW	Oct. 2015	32.0 °N,	4	30.13	7.00	–	–	–	–
P03 NBW		123.6 °E	50	33.73	4.73	–	–	–	–
P03 OSS			52	–	–	–	–	–	–

Table S2. Chl-*a* and DMSP-related factors of ME3, P11 and P03 seawater and oxic surface sediment samples

Sample	Chl-*a* (μg·L^{-1})	DMSPt (nmol·mL^{-1} or g^{-1})	DMSPp > 0.7 μm (nmol·mL^{-1} or g^{-1})	DMSPp > 3 μm (nmol·mL^{-1} or g^{-1})	DMSPp 0.22–3 μm (nmol·mL^{-1} or g^{-1})
ME3 SW	0.35	0.0384 ± 0.0016	0.0317 ± 0.0022	0.0334 ± 0.0043	0.0029 ± 0.0008
ME3 NBW	0.02	0.0115 ± 0.0007	0.0082 ± 0.0010	0.0080 ± 0.0023	0.0016 ± 0.0007
P11 SW	0.07	0.0102 ± 0.0007	0.0077 ± 0.0009	0.0073 ± 0.0020	0.0011 ± 0.0006
P11 NBW	0.01	0.0032 ± 0.0002	0.0022 ± 0.43	0.0020 ± 0.0004	0.0005 ± 0.0002
P03 SW	0.69	0.0884 ±0.0054	0.0838 ± 0.0050	0.0810 ± 0.0082	0.0044 ± 0.0009
P03 NBW	0.07	0.0086 ± 0.0007	0.0060 ± 0.0003	0.0058 ± 0.0007	0.0009 ± 0.0002
P03 OSS	0.11	16.29 ± 1.07		14.19 ± 1.70	

Abbreviation: SW, surface seawater; NBW, near bottom seawater; OSS, oxic surface sediment; PSU, practical salinity unit; DO, dissolved oxygen; Chl-*a*, Chlorophyll *a*; DMSPt, total DMSP; DMSPp, particulate DMSP.

Table S3. Proportion of phytoplankton plastid sequences in total 16S rRNA gene data from amplicon sequencing analysis.

	P03 SW	P03 NBW	P03 SS	ME3 SW	ME3 NBW	P11 SW	P11 NBW
Phytoplanktonic sequences proportion	0.8% ± 0.02%	0.4% ± 0.05%	0.05 ± 0.00%	0.5% ± 0.05%	0.1% ± 0.06%	0.5% ± 0. 1%	0.1% ± 0.02%

Table S4. The phytoplankton community structures of ME3 & P11 derived from flow cytometry.

Phylum/Species/Group*	Phytoplankton abundance（cells/ml）†			
	ME3SW	ME3NBW	P11SW	P11NBW
**Bacillariophyta**	**360.56**	**4.47**	**1.70**	**3.98**
Thalassiosira curviseriata	138.89 (22.2%)	0.21 (3.7%)	-	-
Pseudo-nitzschia delicatissima	127.78 (20.4%)	-	-	-
Skeletonema sp.	35.00 (5.6%)	-	-	-
Leptocylindrus minimus	16.67 (2.7%)	-	-	-
Thalassionema nitzschioides	13.33 (2.1%)	0.43 (7.5%)	-	-
Paralia sulate	-	2.45 (42.7%)	-	3.01 (26.4%)
Plagiogramma sp.	1.11 (0.2%)	0.21 (3.7%)	-	-
Coscinodiscus decrescens	-	0.21 (3.7%)	-	-
Nitzschia panduriformis	-	0.21 (3.7%)	-	-
Chaetoceros compressus	-	-	0.43 (0.8%)	0.22 (1.9%)
**Chlorophyta**	**100.00**	**-**	**0.21**	**-**
Pachysphaera spp.	100.00 (16.0%)	-	-	-
**Dinophyta**	**88.89**	**1.06**	**32.3**	**3.44**
Karlodinium veneficum	35.00 (5.6%)	0.11 (1.9%)	12.1 (23.7%)	1.72 (15.1%)
Scrippsiella trochoidea	23.89 (3.8%)	0.21 (3.7%)	5.53 (10.8)	0.32 (2.8%)
Gyrodinium spirale	-	0.21 (3.7%)	-	-
Akashiwo sanguinea	-	-	2.34 (4.6%)	-
Heterocapsa circularisquama	-	-	1.81 (3.5)	0.32 (2.8%)
Prorocentrum minimum	-	-	1.38 (2.7%)	0.11 (1.0%)
Katodinium glaucum	-	-	1.17 (2.3%)	0.11 (1.0%)

Prorocentrum donghaiense	-	-	0.74 (1.4%)	0.22 (1.9%)
Alexandrium catenella	-	-	0.21 (0.4%)	0.22 (1.9%)
**Cryptophyta**	**34.44**	**-**	**6.7**	**0.22**
Hemiselmis sp.	26.67 (4.3%)	-	2.34 (4.6)	-
Plagioselmis prolonga	-	-	2.98 (5.8%)	0.11 (1.0%)
**Prymnesiophyta**	**30.00**	**0.21**	**10.3**	**3.55**
Coccolithaceae spp.	23.89 (3.8%)	0.21 (3.7%)	1.91 (3.7%)	0.32 (2.8%)
Coccolithaceae (ovoid, species uncertain)	-	-	7.66 (14.9%)	2.80 (24.6%)
Algirosphaera robusta	-	-	0.11 (0.2%)	0.22 (1.9%)
**Euglenophyta**	**11.11**	**-**	**-**	**0.22**
**Chrysophyta**	**1.11**	**-**	**-**	**-**
**Picoeukaryotes‡**	**1.87×10^4**	**0.10×10^4**	**NT$**	**NT$**

*Phytoplankton abundance was listed at phylum level; the top ten most abundant species in each sample were listed (dominant species)

†Percentage indicated contribution of the species to the total phytoplankton abundance in the sample

‡Data were obtained by flow cytometry

$Not determined

Table S5. DMSP concentrations before and after enrichment incubation.

Samples	DMSP concentration (pmol/μg protein)	
	T1C	T1
P03 SW	36.32	494.15
P03 NBW	32.72	232.49
P03 OSS	34.88	118.45

Table S6. qPCR and RT-qPCR analysis of *dsyB* and *mmtN* in P03 seawater and sediment samples. Data were obtained on two or three independent samples (n=2 or n=3) and expressed as mean ± SD.

Sample	***dsyB*** **(copies·mL^{-1} or g^{-1})**	***mmtN*** **(copies·mL^{-1} or g^{-1})**	**16s rRNA (copies·mL^{-1} or g^{-1})**	***rpoB*** **(copies·mL^{-1} or g^{-1})**	**Relative abundance of DMSP producer (%)**
**qPCR**					
SW	2.46E+03 ± 7.86E+01	2.75E+02 ± 2.85E+02	5.13E+05 ± 1.32E+04	ND	0.5
NBW	4.60E+03 ± 1.96E+02	2.13E+01 ± 8.63E-01	7.54E+05 ± 3.07E+04	ND	0.6
OSS	6.68E+06 ± 3.63E+05	1.07E+03 ± 3.91E+02	7.42E+08 ± 3.12E+07	ND	0.9
**RT-qPCR**					
SW	8.17E+00 ± 7.03E+00	1.40E+00 ± 4.20E-01	ND	1.81E+03 ± 3.06E+03	0.5
NBW	8.16E+00 ± 8.67E-02	6.08E+00 ± 4.34E-01	ND	3.75E+02 ± 1.78E+01	3.7
OSS	2.96E+04 ± 5.76E+03	1.99E+04 ± 1.12E+04	ND	1.08E+06 ± 6.07E+04	4.5

ND: not determined.

Table S7. Characteristics of DMSP-producing bacterial isolates.

Strain code	Closest taxonomically related species	Isolation source$	Growth medium*	DMSP production (pmol µg protein^{-1})	Estimated intracellular DMSP concentration (mM)	DsyB and/or MmtN presentation.	Detection Method
ADSW12	*Amorphus suaedae*	P03 SW T1	MB	6.5 ± 0.5	1.0 ± 0.07	DsyB	degenerate PCR
			MBM (minimal, 1 mM NH_4Cl)	N.G.†	N.G.		
ADSW13	*Amorphus suaedae*	P03 SW T1	MB	5.4 ± 0.1	0.9 ± 0.02	DsyB	degenerate PCR
			MBM (minimal, 1 mM NH_4Cl)	N.G.	N.G.		
ADSW14	*Amorphus suaedae*	P03 SW T1	MB	6.6 ± 0.3	1.0 ± 0.05	DsyB	degenerate PCR
			MBM (minimal, 1 mM NH_4Cl)	N.G.	N.G.		
ADSW24	*Amorphus suaedae*	P03 SW T1	MB	6.0 ± 0.4	0.9 ± 0.06	DsyB	degenerate PCR
			MBM (minimal, 1 mM NH_4Cl)	N.G.	N.G.		
ADSW25-1	*Amorphus suaedae*	P03 SW T1	MB	10.4 ± 0.6	1.6 ± 0.1	DsyB	degenerate PCR
			MBM (minimal, 1 mM NH_4Cl)	N.G.	N.G.		
ADSW29	*Acuticoccus yangtzensis*	P03 SW T1	MB	89.9 ± 11.5	14.1 ± 1.8	DsyB	degenerate PCR
			MBM (minimal, 1 mM NH_4Cl)	79.5 ± 5.2	12.4 ± 0.8		
ADBW18	*Pelagibaca bermudensis*	P03 NBW T1	MB	55.3 ± 1.3	8.6 ± 0.2	DsyB	degenerate PCR

			MBM (minimal, 1 mM NH_4Cl)	9.7 ± 0.7	1.5 ± 0.1		
ADSS11-1	*Stappia taiwanensis*	P03 OSS T1	MB	7.9 ± 0.8	1.2 ± 0.1	DsyB	degenerate PCR
			MBM (minimal, 1 mM NH_4Cl)	22.9 ± 0.9	3.6 ± 0.1		
ADSS19	*Stappia taiwanensis*	P03 OSS T1	MB	9.6 ± 0.8	1.5 ± 0.1	DsyB	degenerate PCR
			MBM (minimal, 1 mM NH_4Cl)	25.3 ± 0.1	4.0 ± 0.02		
ADSS20	*Stappia taiwanensis*	P03 OSS T1	MB	12.7 ± 1.1	2.0 ± 0.2	DsyB	degenerate PCR
			MBM (minimal, 1 mM NH_4Cl)	25.4 ± 1.3	4.0 ± 0.2		
ADSS28	*Stappia taiwanensis*	P03 OSS T1	MB	14.5 ± 1.8	2.3 ± 0.3	DsyB	degenerate PCR
			MBM (minimal, 1 mM NH_4Cl)	23.0 ± 0.6	3.6 ± 0.1		
AESS21	*Labrenzia suaedae*	P03 OSS T1	MB	131.1 ± 2.6	20.5 ± 0.4	DsyB	degenerate PCR & genome sequencing
			MBM (minimal, 1 mM NH_4Cl)	6.1 ± 0.1	1.0 ± 0.02		
AESS44	*Labrenzia suaedae*	P03 OSS T1	MB	169.2 ± 5.0	26.5 ± 0.8	DsyB	degenerate PCR
			MBM (minimal, 1 mM NH_4Cl)	6.0 ± 0.3	0.9 ± 0.05		
AESS46	*Labrenzia suaedae*	P03 OSS T1	MB	186.0 ± 22.5	29.1 ± 3.5	DsyB	degenerate PCR
			MBM (minimal, 1 mM NH_4Cl)	6.1 ± 0.02	1.0 ± 0.003		
AESS41	*Marinobacter lipolyticus*	P03 OSS T1	MB	6.3 ± 0.3	1.0 ± 0.05	N.D.	degenerate PCR & genome sequencing
			MBM (minimal, 1	N.D.‡	N.D.		

			mM NH_4Cl)				
AESS51-2	*Marinobacter adhaerens*	P03 OSS T1	MB	1.9 ± 0.1	0.3 ± 0.02	N.D.	degenerate PCR
			MBM (minimal, 1 mM NH_4Cl)	N.D.	N.D.		
BDSW03	*Pseudooceanicola antarcticus*	P03 SW T1	MB	148.8 ± 23.6	23.3 ± 3.7	DsyB	degenerate PCR
			MBM (minimal, 1 mM NH_4Cl)	36.5 ± 7.2	5.7 ± 1.1		
BDSW11	*Pseudooceanicola antarcticus*	P03 SW T1	MB	189.6 ± 4.3	29.7 ± 0.7	DsyB	degenerate PCR
			MBM (minimal, 1 mM NH_4Cl)	29.6 ± 1.1	4.6 ± 0.2		
BDSW15	*Pseudooceanicola antarcticus*	P03 SW T1	MB	191.3 ± 6.6	29.9 ± 1.0	DsyB	degenerate PCR
			MBM (minimal, 1 mM NH_4Cl)	30.9 ± 3.5	4.8 ± 0.5		
BDSW19	*Pseudooceanicola antarcticus*	P03 SW T1	MB	150.3 ± 29.0	23.5 ± 4.5	DsyB	degenerate PCR
			MBM (minimal, 1 mM NH_4Cl)	31.3 ± 3.0	4.9 ± 0.5		
BDSW30	*Pseudooceanicola antarcticus*	P03 SW T1	MB	196.3 ± 22.3	30.7 ± 3.5	DsyB	degenerate PCR
			MBM (minimal, 1 mM NH_4Cl)	17.8 ± 0.4	2.8 ± 0.1		
BDBW05	*Pelagibaca bermudensis*	P03 NBW T1	MB	28.7 ± 2.9	4.5 ± 0.5	DsyB	degenerate PCR

			MBM (minimal, 1 mM NH_4Cl)	11.2 ± 0.5	1.8 ± 0.1		
BDBW16	*Pelagibaca bermudensis*	P03 NBW T1	MB	32.0 ± 4.4	5.0 ± 0.7	DsyB	degenerate PCR
			MBM (minimal, 1 mM NH_4Cl)	9.5 ± 0.3	1.5 ± 0.05		
BDSS02	*Sulfitobacter dubis*	P03 OSS T1	MB	2.9 ± 0.04	0.5 ± 0.007	DsyB	genome sequencing
			MBM (minimal, 1 mM NH_4Cl)	N.G.	N.G.		
BDSS04	*Sulfitobacter dubis*	P03 OSS T1	MB	2.8 ± 0.4	0.4 ± 0.06	N.D.	degenerate PCR
			MBM (minimal, 1 mM NH_4Cl)	N.G.	N.G.		
BDSS12	*Labrenzia aggregata*	P03 OSS T1	MB	44.0 ± 1.3	6.9 ± 0.2	DsyB	degenerate PCR
			MBM (minimal, 1 mM NH_4Cl)	20.2 ± 0.8	3.2 ± 0.1		
BDSS19	*Sulfitobacter dubis*	P03 OSS T1	MB	3.2 ± 0.4	0.5 ± 0.06	N.D.	degenerate PCR
			MBM (minimal, 1 mM NH_4Cl)	N.G.	N.G.		
BEBW06	*Thalassospira profundimaris*	P03 NBW T1	MB	1.2 ± 0.1	0.2 ± 0.02	MmtN	degenerate PCR
			MBM (minimal, 1 mM NH_4Cl)	85.5 ± 6.3	13.4 ±1.0		
BEBW11	*Thalassospira profundimaris*	P03 NBW T1	MB	1.1 ± 0.3	0.2 ± 0.04	MmtN	degenerate PCR
			MBM (minimal, 1 mM NH_4Cl)	81.6 ± 1.6	12.8 ± 0.2		
BEBW19	*Thalassospira*	P03 NBW	MB	1.3 ± 0.1	0.2 ± 0.02	MmtN	degenerate PCR

	profundimaris	T1					
			MBM (minimal, 1 mM NH_4Cl)	86.0 ± 2.1	13.5 ± 0.3		
BEBW25-2	*Thalassospira profundimaris*	P03 NBW T1	MB	1.2 ± 0.01	0.2 ± 0.001	MmtN	degenerate PCR
			MBM (minimal, 1 mM NH_4Cl)	81.9 ± 0.7	12.8 ± 0.1		
BEBW28	*Thalassospira tepidiphila*	P03 NBW T1	MB	8.6 ± 0.2	1.4 ± 0.04	MmtN	degenerate PCR & genome sequencing
			MBM (minimal, 1 mM NH_4Cl)	339.9 ± 2.8	53.2 ± 0.4		
BEBW31-1	*Thalassospira profundimaris*	P03 NBW T1	MB	1.2 ± 0.04	0.2 ± 0.01	MmtN	degenerate PCR
			MBM (minimal, 1 mM NH_4Cl)	84.5 ± 1.0	13.2 ± 0.2		
BEBW31-2	*Thalassospira profundimaris*	P03 NBW T1	MB	1.3 ± 0.1	0.2 ± 0.02	MmtN	degenerate PCR
			MBM (minimal, 1 mM NH_4Cl)	391.6 ± 27.1	61.3 ± 4.2		
BEBW32	*Thalassospira tepidiphila*	P03 NBW T1	MB	8.0 ± 0.1	1.3 ± 0.02	MmtN	degenerate PCR
			MBM (minimal, 1 mM NH_4Cl)	336.6 ± 9.4	52.6 ± 1.5		
BESS04	*Labrenzia suaedae*	P03 OSS T1	MB	73.3 ± 0.4	11.5 ± 0.1	DsyB.	degenerate PCR
			MBM (minimal, 1 mM NH_4Cl)	5.0 ± 0.03	0.8 ± 0.004		
BESS05	*Labrenzia suaedae*	P03 OSS T1	MB	84.7 ± 2.1	13.2 ± 0.3	DsyB	degenerate PCR

			MBM (minimal, 1 mM NH_4Cl)	4.9 ± 0.1	0.8 ± 0.01		
BESS10	*Labrenzia suaedae*	P03 OSS T1	MB	136.1 ± 6.0	21.3 ± 0.9	N.D.	degenerate PCR
			MBM (minimal, 1 mM NH_4Cl)	4.7 ± 0.1	0.7 ± 0.02		
BESS13	*Labrenzia suaedae*	P03 OSS T1	MB	77.8 ± 2.2	12.2 ± 0.3	DsyB	degenerate PCR
			MBM (minimal, 1 mM NH_4Cl)	4.7 ± 0.3	0.7 ± 0.04		
BESS15	*Labrenzia suaedae*	P03 OSS T1	MB	72.6 ± 4.0	11.4 ± 0.6	DsyB	degenerate PCR
			MBM (minimal, 1 mM NH_4Cl)	4.4 ± 0.3	0.7 ± 0.04		
BESS17	*Labrenzia suaedae*	P03 OSS T1	MB	133.7 ± 3.8	20.9 ± 0.6	DsyB	degenerate PCR of
			MBM (minimal, 1 mM NH_4Cl)	14.0 ± 1.6	2.2 ± 0.2		
BESS24	*Labrenzia alexandrii*	P03 OSS T1	MB	47.6 ± 0.7	7.4 ± 0.1	DsyB	degenerate PCR of *dsyB*
			MBM (minimal, 1 mM NH_4Cl)	11.7 ± 0.4	1.8 ± 0.06		
BESS25	*Labrenzia suaedae*	P03 OSS T1	MB	130.4 ± 0.03	20.4 ± 0.01	DsyB	degenerate PCR
			MBM (minimal, 1 mM NH_4Cl)	11.1 ± 0.01	1.7 ± 0.002		
DSW02	*Labrenzia aggregata*	P03 SW T0	MB	68.0 ± 7.3	10.6 ± 1.1	DsyB	degenerate PCR
			MBM (minimal, 1 mM NH_4Cl)	28.6 ± 0.06	4.5 ± 0.009		
DSW17	*Poseidonocella pacifica*	P03 SW T0	MB	49.0 ± 3.9	7.7 ± 0.6	DsyB	degenerate PCR
			MBM (minimal, 1 mM NH_4Cl)	33.8 ± 1.3	5.3 ± 0.2		

DSW18	*Marinobacter salarius*	P03 SW T0	MB	1.4 ± 0.3	0.2 ± 0.05	N.D.	degenerate PCR & genome sequencing
			MBM (minimal, 1 mM NH_4Cl)	N.G.	N.G.		
EBW16	*Oceanicola litoreus*	P03 NBW T0	MB	4.3 ± 0.1	0.7 ± 0.02	DsyB	degenerate PCR & genome sequencing
			MBM (minimal, 1 mM NH_4Cl)	N.D.	N.D.		
ESS08	*Bacillus boroniphilus*	P03 OSS T0	MB	0.9 ± 0.2	0.1 ± 0.03	N.D.	degenerate PCR & genome sequencing
			MBM (minimal, 1 mM NH_4Cl)	N.G.	N.G.		
ZYFB032	*Pelagibaca bermudensis*	P11 SW T0	MB	86.9 ± 2.2	13.6 ± 0.3	DsyB	degenerate PCR
			MBM (minimal, 10 mM NH_4Cl)	3.4 ± 0.1	0.5 ± 0.02		
			MBM (minimal, 1 mM NH_4Cl)	10.7 ± 1.1	1.7 ± 0.2		
			MBM (minimal, 0.5 mM NH_4Cl)	91.0 ± 3.6	14.2 ± 0.6		
LZB033	*Labrenzia aggregata*	ME3 SW T0	MB	62.4 ± 0.6	9.8 ± 0.1	DsyB	degenerate PCR & genome sequencing
			MBM (minimal, 10 mM NH_4Cl)	4.9 ± 0.3	0.8 ± 0.05		
			MBM (minimal, 1 mM NH_4Cl)	25.7 ± 0.3	4.0 ± 0.1		
			MBM (minimal, 0.5	99.8 ± 1.2	15.6 ± 0.2		

			mM NH_4Cl)				
LZB062	*Pseudooceanicola nanhaiensis*	ME3 SW T0	MB	43.6 ± 0.6	6.8 ± 0.1	DsyB	degenerate PCR
			MBM (minimal, 10 mM NH_4Cl)	8.4 ± 0.1	1.3 ± 0.02		
			MBM (minimal, 1 mM NH_4Cl)	132.6 ± 12.2	20.7 ± 1.9		
			MBM (minimal, 0.5 mM NH_4Cl)	3051.2 ± 52.9	477.2 ± 8.3		
LZD001	*Pelagibaca bermudensis*	ME3 NBW T0	MB	63.8 ± 3.5	10.0 ± 0.5	DsyB	degenerate PCR
			MBM (minimal, 10 mM NH_4Cl)	5.0 ± 0.4	0.8 ± 0.1		
			MBM (minimal, 1 mM NH_4Cl)	11.5 ± 0.7	1.8 ± 0.1		
			MBM (minimal, 0.5 mM NH_4Cl)	145.2 ± 0.8	22.7 ± 0.1		
LZD012	*Halomonas saccharevitans*	ME3 NBW T0	MB	1.4 ± 0.2	0.2 ± 0.02	N.D.	degenerate PCR & genome sequencing
			MBM (minimal, 10 mM NH_4Cl)	N.D.	N.D.		
			MBM (minimal, 1 mM NH_4Cl)	N.D.	N.D.		
			MBM (minimal, 0.5 mM NH_4Cl)	N.D.	N.D.		
LZD019	*Pelagibaca bermudensis*	ME3 NBW T0	MB	67.3 ± 0.1	10.5 ± 0.01	DsyB	degenerate PCR

			MBM (minimal, 10 mM NH_4Cl)	5.1 ± 0.1	0.8 ± 0.01		
			MBM (minimal, 1 mM NH_4Cl)	10.8 ± 0.4	1.7 ± 0.06		
			MBM (minimal, 0.5 mM NH_4Cl)	130.3 ± 2.7	20.4 ± 0.4		
LZD031	*Pelagibaca bermudensis*	ME3 NBW T0	MB	80.6 ± 5.0	12.6 ± 0.8	DsyB	degenerate PCR
			MBM (minimal, 10 mM NH_4Cl)	5.1 ± 0.1	0.8 ± 0.02		
			MBM (minimal, 1 mM NH_4Cl)	11.6 ± 0.2	1.8 ± 0.03		
			MBM (minimal, 0.5 mM NH_4Cl)	162.2 ± 2.1	25.4 ± 0.3		
LZD033	*Pelagibaca bermudensis*	ME3 NBW T0	MB	66.9 ± 2.2	10.5 ± 0.3	DsyB	degenerate PCR
			MBM (minimal, 10 mM NH_4Cl)	4.6 ± 0.3	0.7 ± 0.05		
			MBM (minimal, 1 mM NH_4Cl)	9.2 ± 0.1	1.4 ± 0.02		
			MBM (minimal, 0.5 mM NH_4Cl)	165.0 ± 1.7	25.8 ± 0.3		
LZD040	*Pelagibaca bermudensis*	ME3 NBW T0	MB	69.1 ± 0.1	10.8 ± 0.02	DsyB	degenerate PCR
			MBM (minimal, 10 mM NH_4Cl)	5.5 ± 0.5	0.9 ± 0.08		
			MBM (minimal, 1	9.7 ± 0.1	1.5 ± 0.01		

			mM NH_4Cl)				
			MBM (minimal, 0.5 mM NH_4Cl)	120.3 ± 3.2	18.8 ± 0.5		
LZD042	*Pelagibaca bermudensis*	ME3 T0	NBW	MB	72.0 ± 1.0	11.3 ± 0.2	DsyB	degenerate PCR
			MBM (minimal, 10 mM NH_4Cl)	10.0 ± 0.5	1.6 ± 0.08		
			MBM (minimal, 1 mM NH_4Cl)	19.9 ± 2.1	3.1 ± 0.3		
			MBM (minimal, 0.5 mM NH_4Cl)	218.2 ± 5.5	34.1 ± 0.9		
LZD043	*Pelagibaca bermudensis*	ME3 T0 NBW	MB	53.7 ± 1.7	8.4 ± 0.3	DsyB	degenerate PCR
			MBM (minimal, 10 mM NH_4Cl)	N.D.	N.D.		
			MBM (minimal, 1 mM NH_4Cl)	22.3 ± 3.9	3.5 ± 0.6		
			MBM (minimal, 0.5 mM NH_4Cl)	125.1 ± 2.1	19.6 ± 0.3		
LZD044	*Pelagibaca bermudensis*	ME3 T0 NBW	MB	76.4 ± 0.1	12.0 ± 0.02	DsyB	degenerate PCR
			MBM (minimal, 10 mM NH_4Cl)	4.4 ± 0.4	0.7 ± 0.05		
			MBM (minimal, 1 mM NH_4Cl)	10.4 ± 0.3	1.6 ± 0.05		
			MBM (minimal, 0.5 mM NH_4Cl)	163.7 ± 3.4	25.6 ± 0.5		

LZD059	*Marinobacter algicola*	ME3 T0	NBW	MB	1.7 ± 0.1	0.3 ± 0.02	N.D.	degenerate PCR
				MBM (minimal, 10 mM NH_4Cl)	N.D.	N.G.		
				MBM (minimal, 1 mM NH_4Cl)	N.D.	N.G.		
				MBM (minimal, 0.5 mM NH_4Cl)	N.D.	N.G.		
LZD060	*Marinobacter salsuginis*	ME3 T0	NBW	MB	2.0 ± 0.5	0.3 ± 0.07	N.D.	degenerate PCR
				MBM (minimal, 10 mM NH_4Cl)	N.D.	N.G.		
				MBM (minimal, 1 mM NH_4Cl)	N.D.	N.G.		
				MBM (minimal, 0.5 mM NH_4Cl)	N.D.	N.G.		

The values for DMSP production and estimated intracellular DMSP concentration are the averages of three biological replicates with the s.d. being shown.

$“SW’, ‘NBW’ and “OSS” present surface seawater, near bottom seawater and oxic surface sediment, respectively; T0 and T1 present natural and DMSP production process enriching samples, respectively.

*No added methylated sulfur compounds unless stated otherwise; 2 mM succinate + 2 mM glucose + 2 mM sucrose + 2 mM pyruvate + 2 mM glycerol were used as carbon source mixture for MBM media.

†N.G., no growth.

‡N.D., not detected.

Table S8. MAGs recovered from the metagenomic data.

MAGs	Sample	Taxonnamy	Completeness	Contamination	DMSP biosynthesis gene
MAG1	ME3 SW T0	d__Bacteria.p__Proteobacteria.c__Alphaproteobacteria.o__Rhodobacterales.f__Rhodobacteraceae	99.7	0.55	*dsyB*
MAG2	P03 NBW T1	d__Bacteria.p__Proteobacteria.c__Alphaproteobacteria.o__Rhodospirillales.f__Rhodospirillaceae	100	0	*mmtN*
MAG3	P03 OSS T1	d__Bacteria.p__Proteobacteria.c__Alphaproteobacteria	99.29	0.85	*dsyB*
MAG4	P03 SW T1	d__Bacteria.p__Proteobacteria.c__Alphaproteobacteria	90.1	2.36	*dsyB*
MAG5	P03 SW T1C	d__Bacteria.p__Proteobacteria.c__Alphaproteobacteria	96.55	4.34	*dsyB*
MAG6	P03 SW T1C	d__Bacteria.p__Proteobacteria.c__Gammaproteobacteria.o__Oceanospirillales.f__Halomonadaceae	98.71	0.43	
MAG7	P03 SW T1C	d__Bacteria.p__Bacteroidetes.c__Cytophagia.o__Cytophagales	99.26	0.63	
MAG8	P03 SW T1C	d__Bacteria.p__Proteobacteria.c__Gammaproteobacteria.o__Oceanospirillales.f__Halomonadaceae	96.1	1.08	
MAG9	P03 SW T1C	d__Bacteria.p__Planctomycetes.c__Planctomycetacia.o__Planctomycetales.f__Planctomycetaceae	96.55	4.34	
MAG10	P03 SW T1C	d__Bacteria.p__Proteobacteria.c__Gammaproteobacteria.o__Alteromonadales.f__Alteromonadaceae.g__Alteromonas.s__macleodii	87.55	1.67	
MAG11	P03 SW T1C	d__Bacteria.p__Bacteroidetes	100	0	
MAG12	P03 SW T1C	d__Bacteria.p__Proteobacteria.c__Alphaproteobacteria.o__Rhodospirillales.f__Rhodospirillaceae	94.98	1.49	
MAG13	P03 SW T1	d__Bacteria.p__Proteobacteria.c__Alphaproteobacteria.o__Rhodobacterales.f__Rhodobacteraceae	99.6	1.51	
MAG14	P03 SW T1	d__Bacteria.p__Proteobacteria.c__Alphaproteobacteria.o__Rhodobacterales.f__Hyphomonadaceae	99.68	1.3	

MAG15	P03 SW T1	d__Bacteria.p__Proteobacteria.c__Gammaproteobacteria.o__Oceanospirillales.f__Halomonadaceae	94.7	1.03
MAG16	P03 SW T1	d__Bacteria.p__Proteobacteria.c__Alphaproteobacteria.o__Rhodospirillales.f__Rhodospirillaceae	97.64	0
MAG17	P03 SW T0	d__Bacteria.p__Proteobacteria.c__Gammaproteobacteria	84.88	1.51
MAG18	P03 SW T0	d__Bacteria.p__Verrucomicrobia.c__Verrucomicrobiae.o__Verrucomicrobiales.f__Verrucomicrobiaceae	95.89	3.63
MAG19	P03 SW T0	d__Bacteria.p__Verrucomicrobia.c__Verrucomicrobiae.o__Verrucomicrobiales.f__Verrucomicrobiaceae	96.6	0.68
MAG20	P03 SW T0	d__Bacteria.p__Proteobacteria.c__Alphaproteobacteria	89.14	2.23
MAG21	P03 OSS T1C	d__Bacteria.p__Bacteroidetes	98.68	0.85
MAG22	P03 OSS T1C	d__Bacteria.p__Proteobacteria.c__Alphaproteobacteria.o__Rhodobacterales.f__Hyphomonadaceae	99.38	1.04
MAG23	P03 OSS T1C	d__Bacteria.p__Proteobacteria.c__Alphaproteobacteria.o__Rhodobacterales.f__Hyphomonadaceae	89.26	1.73
MAG24	P03 OSS T1C	d__Bacteria.p__Proteobacteria.c__Gammaproteobacteria.o__Oceanospirillales	97.39	1.52
MAG25	P03 OSS T1C	d__Bacteria.p__Proteobacteria.c__Gammaproteobacteria.o__Vibrionales.f__Vibrionaceae.g__Vibrio	95.27	0.87
MAG26	P03 OSS T1C	d__Bacteria.p__Bacteroidetes.c__Flavobacteria.o__Flavobacteriales.f__Flavobacteriaceae	98.42	0.25
MAG27	P03 OSS T1C	d__Bacteria.p__Proteobacteria.c__Gammaproteobacteria.o__Aeromonadales	90.09	0.54
MAG28	P03 OSS T1	d__Bacteria.p__Bacteroidetes	90.44	1.64
MAG29	P03 OSS T1	d__Bacteria.p__Bacteroidetes.c__Flavobacteria.o__Flavobacteriales.f__Flavobacteriaceae	93.15	3.12
MAG30	P03 OSS T1	d__Bacteria.p__Proteobacteria.c__Gammaproteobacteria.o__Alteromon	99.84	0.24

		adales.f__Alteromonadaceae.g__Alteromonas.s__macleodii		
MAG31	P03 OSS T1	d__Bacteria.p__Proteobacteria.c__Gammaproteobacteria	99.9	1.4
MAG32	P03 OSS T1	d__Bacteria.p__Bacteroidetes.c__Flavobacteria.o__Flavobacteriales.f__ Flavobacteriaceae	97.3	5.22
MAG33	P03 OSS T1	d__Bacteria.p__Bacteroidetes	98.51	0.99
MAG34	P03 OSS T1	d__Bacteria.p__Proteobacteria.c__Gammaproteobacteria.o__Alteromon adales.f__Idiomarinaceae.g__Idiomarina	98.65	0.39
MAG35	P03 OSS T1	d__Bacteria.p__Proteobacteria.c__Gammaproteobacteria.o__Oceanospir illales	96.18	4.74
MAG36	P03 OSS T1	d__Bacteria.p__Bacteroidetes	98.99	0.52
MAG37	P03 OSS T1	d__Bacteria.p__Proteobacteria.c__Gammaproteobacteria.o__Oceanospir illales.f__Halomonadaceae	99.29	0.85
MAG38	P03 OSS T1	d__Bacteria.p__Proteobacteria.c__Gammaproteobacteria.o__Alteromon adales.f__Alteromonadaceae.g__Marinobacter	95.49	0.68
MAG39	P03 OSS T1	d__Bacteria.p__Proteobacteria.c__Alphaproteobacteria	93.29	0.7
MAG40	P03 OSS T1	d__Bacteria.p__Proteobacteria.c__Alphaproteobacteria.o__Rhodobacter ales.f__Rhodobacteraceae	99.54	0.83
MAG41	P03 OSS T1	d__Bacteria.p__Proteobacteria.c__Gammaproteobacteria.o__Vibrionales .f__Vibrionaceae.g__Vibrio	97.78	1.12
MAG42	P03 NBW T1C	d__Bacteria.p__Proteobacteria.c__Gammaproteobacteria.o__Oceanospir illales	99.14	0.21
MAG43	P03 NBW T1C	d__Bacteria.p__Proteobacteria.c__Gammaproteobacteria.o__Oceanospir illales	99.14	0.64
MAG44	P03 NBW T1C	d__Bacteria.p__Proteobacteria.c__Gammaproteobacteria.o__Oceanospir illales.f__Halomonadaceae	98.37	1.51
MAG45	P03 NBW T1C	d__Bacteria.p__Proteobacteria.c__Gammaproteobacteria.o__Alteromon adales.f__Alteromonadaceae.g__Alteromonas.s__macleodii	99.55	1.53

MAG46	P03 NBW T1C	d__Bacteria.p__Proteobacteria.c__Gammaproteobacteria.o__Oceanospirillales.f__Halomonadaceae	90.04	1.36
MAG47	P11 NBW T0	d__Bacteria.p__Proteobacteria.c__Gammaproteobacteria.o__Oceanospirillales.f__Halomonadaceae	94.85	3.1
MAG48	P11 NBW T0	d__Bacteria.p__Proteobacteria.c__Gammaproteobacteria	98.07	1.27
MAG49	P11 NBW T0	d__Bacteria.p__Actinobacteria.c__Actinobacteria	97.09	0
MAG50	ME3 SW T0	d__Bacteria.p__Actinobacteria.c__Actinobacteria	91.25	3.79

Table S9. Basic information of metagenomic data.

Sample	Clean reads	Contig Number	Assembly Length (bp)	N50 (bp)	N75(bp)	CDS number
P11SWT0	18,474,140	94,043	68,751,933	699	576	899,663
P11NBWT0	17,723,312	28,681	21,594,513	723	582	279,816
ME3SWT0	18,962,344	53,042	40,923,951	747	588	560,755
ME3NBWT0	12,614,636	57,887	43,451,151	717	579	670,752
P03SWT0	14,570,444	39,499	49,367,974	1,359	699	604,531
P03NBWT0	20,865,496	947	673,425	696	582	31,750
P03OSST0	8,960,848	3,320	2,422,894	661	561	385,897
P03SWT1	20,746,884	16,633	41,182,818	32,366	1,442	105,705
P03NBWT1	21,760,928	13,566	39,374,403	12,644	1,916	82,333
P03OSST1	21,305,296	73,162	143,688,576	3,604	1,128	393,315
P03SWT1C	22,489,728	29,829	77,961,891	13,605	1,613	171,540
P03NBWT1C	22,230,216	6,494	15,074,973	90,873	1,189	50,053
P03OSST1C	22,015,280	31,582	80,915,269	7,185	1,690	186,178

Table S10. Composition of normal or modified MBM medium

Basal Media* (pH 7.5)	Tris	34.61 g
	K_2HPO_4	0.17 g
	H_2O	1000 ml
1 M NH_4Cl stock solution	NH_4Cl	5.35 g
	H_2O	100 ml
FeEDTA stock solution*	FeEDTA	0.05 g
	H_2O	100 ml
0.5 M L-methionine (L-Met) stock solution†	L-Met	7.46 g
	H_2O	100 ml
Carbon source stock solution†	Disodium succinate hexahydrate	54 g
	Glucose	36.3 g
	Sucrose	68.4 g
	Sodium pyruvate	22 g
	Glycerol	14.6 g
	H_2O	985.4 ml
Normal MBM (pH 7.0)	Sea Salts (Sigma-Aldrich, UK)	35 g
	Basal Media	250 ml
	FeEDTA stock	50 ml
	NH_4Cl stock	10 ml
	Vitamin supplement stock†§	10 ml
	Carbon source stock solution	10 ml
	H_2O	670 ml
For modified MBM (pH 7.0)	Sea Salts (Sigma-Aldrich, UK)	50 g
	Basal Media	250 ml
	FeEDTA stock	50 ml
	NH_4Cl stock	1 ml
	L-Met stock	1 ml
	Vitamin supplement stock†§	10 ml
	Carbon source stock solution	10 ml
	H_2O	678 ml

*Autoclaving to sterilize

†Filtering to sterilize

§Balch et al., 1979

Table S11. Reference protein sequences of the functionally ratified DMSP degradation and biosynthesis enzymes

Protein	Source	NCBI Accession Number	Reference
DsyB	*Labrenzia aggregata* IAM 12614	EAV42226	Curson et al., 2017
	Pseudooceanicola batsensis HTCC2597	EAQ04968	Curson *et al*., 2017
	Pelagibaca bermudensis HTCC2601	EAU45958	Curson *et al*., 2017
	Amorphus coralli DSM 19760	WP_018697905	Curson *et al*., 2017
MmtN	*Thalassospira profundimaris* PB8B	OAZ15224	Williams *et al*., 2019
	Novosphingobium sp. MBES04	GAM03459	Williams *et al*., 2019
	Roseovarius indicus B108	KRS18724	Williams *et al*., 2019
	Nocardiopsis chromatogenes YIM 90109	WP_017624909	Williams *et al*., 2019
	Streptomyces mobaraensis DSM 40847	EME99407	Williams *et al*., 2019
DSYB	*Prymnesium parvum* CCAP946/6	N.A.*	Curson et al., 2018
	Chrysochromulina tobin CCMP291	KOO32714	Curson *et al*., 2018
	Lingulodinium polyedrum CCMP1936	N.A.	Curson *et al*., 2018
	Alexandrium tamarense ATSP1-B	N.A.	Curson *et al*., 2018
	Acropora cervicornis	N.A.	Curson *et al*., 2018
	Fragilariopsis cylindrus CCMP1102	OEU17621	Curson *et al*., 2018
	Symbiodinium microadriaticum CCMP2467	OLQ07620	Curson *et al*., 2018
TpMMT	*Thalassiosira pseudonana* CCMP1335	Tp23128	Kageyama et al., 2018
DmdA	*Ruegeria pomeroyi* DSS-3	AAV95190	Howard et al., 2006
	Pelagibacter ubique HTCC1062	WP_011281570	Howard *et al*., 2006
	Dinoroseobacter shibae DFL 12	WP_012178987	Howard et al., 2008
	marine gammaproteobacterium HTCC2080	WP_007233625	Howard *et al*., 2008
	Candidatus Pelagibacter sp. HTCC7211	WP_008546106	Howard et al., 2011
	Candidatus Puniceispirillum marinum IMCC1322	WP_013044947	Howard *et al*., 2011
DddD	*Marinomonas* sp. MWYL1	ABR72937	Todd et al., 2007

	Oceanimonas doudoroffii	AEQ39135	Curson et al., 2012
	Psychrobacter sp. J466	ACY02894	Curson *et al*., 2010
	Halomonas sp. HTNK1	ACV84065	Todd et al., 2010
	Sinorhizobium fredii NGR234	AAQ87407	Todd et al., 2007
	Burkholderia ambifaria AMMD	WP_011659284	Todd *et al*., 2007
	Pseudomonas sp. J465	ACY01992	Curson *et al*., 2010
	Sagittula stellata E-37	EBA08656	Curson et al., 2011a
DddL	*Sulfitobacter* sp. EE-36	ADK55772	Curson et al., 2008
	Rhodobacter sphaeroides 2.4.1	YP_351475	Curson *et al*., 2008
	Labrenzia aggregata LZB033	KP639184	Curson *et al*., 2017
	Ahrensia marina LZD062	KP639183	Curson et al., 2017
DddP	*Roseovarius nubinhibens* ISM	EAP77700	Todd et al., 2009
	Ruegeria pomeroyi DSS-3	WP_044029245	Todd et al., 2011
	Phaeobacter inhibens DSM 17395	AFO91571	Burkhardt et al., 2017
	Oceanimonas doudoroffii DSM 7028	AEQ39091	Curson *et al*., 2012
	Oceanimonas doudoroffii DSM 7028	AEQ39103	Curson *et al*., 2012
	Aspergillus oryzae RIB40	BAE62778	Todd et al., 2009
	Fusarium graminearum PH-1	XP_389272	Todd *et al*., 2009
	Candidatus Puniceispirillum marinum	WP_013046297	Choi et al., 2015
	Ruegeria lacuscaerulensis ITI-1157	WP_005982191	Wang et al., 2015
DddQ	*Ruegeria pomeroyi* DSS-3	WP_011047333	Todd *et al*., 2011
	Roseovarius nubinhibens ISM	EAP76002	Todd *et al*., 2011
	Roseovarius nubinhibens ISM	EAP76001	Todd *et al*., 2011
	Ruegeria lacuscaerulensis ITI-1157	WP_005978225	Li et al., 2014
	GOS databases	ECW91654	Todd *et al*., 2011
	GOS databases	EBP74803	Todd *et al*., 2011

	GOS databases	ECX82089	Todd *et al*., 2011
DddW	*Ruegeria pomeroyi* DSS-3	AAV93771	Todd et al., 2012
DddY	*Alcaligenes faecalis* M3A	ADT64689	Souza et al., 1995; Curson et al., 2011a; Lei et al., 2018
	Shewanella putrefaciens CN-32	ABP77243	Curson *et al*., 2011b; Lei *et al*., 2018
	Desulfovibrio acrylicus	SHJ73420	Van Der Maarel et al., 1996; Lei *et al*., 2018
	Ferrimonas kyonanensis DSM 18153	WP_028114584	Lei *et al*., 2018
	Acinetobacter bereziniae	ENV21217	Li et al., 2017
DddK	*Candidatus* Pelagibacter ubique HTCC1062	AAZ21215	Sun et al., 2016
	Alphaproteobacterium HIMB5	AFS47241	Sun *et al*., 2016
	Candidatus Pelagibacter ubique HTCC9022	WP_028037226	Sun *et al*., 2016
Alma1	*Emiliania huxleyi* CCMP1516	XP_005784450	Alcolombri et al., 2015
	Emiliania huxleyi CCMP1516	XP_005763983	Alcolombri *et al*., 2015
	Symbiodinium sp. clade D	P0DN22	Alcolombri *et al*., 2015

*N.A., not available.

Table S12. Oligonucleotide primers used in this study.

Primer name	Sequence (5' to 3')	Use	Reference
27F<br>1492R	AGAGTTTGATCCTGGCTCAG<br>GGTTACCTTGTTACGACTT	Amplification of cultivated strain's 16S rRNA gene	DeLong, 1992; Lane et al., 1985
515F<br>806R	GTGCCAGCMGCCGCGG<br>GGACTACHVGGGTWTCTAAT	Preparing partial 16S rRNA genes amplicon for pyrosequencing	Caporaso et al., 2012
Eub338F<br>Eub518R	ACTCCTACGGGAGGCAGCAG<br>ATTACCGCGGCTGCTGG	qPCR of 16S rRNA gene	Yin et al., 2013
dsyBF<br>dsyBR	CATGGGSTCSAAGGCSCTKTT<br>GCAGRTARTCGCCGAAATCGTA	Degenerate primers for *dsyB* detection in DMSP producing bacterial isolates and qPCR of *dsyB*	Williams *et al.*, 2019
mmtNF<br>mmtNR	CCGAGGTGGTCATGAAYTTYGG<br>CCGAGGTGGTCATGAAYTTYGG	Degenerate primers for *mmtN* detection in DMSP producing bacterial isolates and qPCR and RT-qPCR of *mmtN*	Williams *et al.*, 2019

Fig. S1 Alpha- and beta diversity analyses from 16S rRNA amplicon sequencing data. All samples were rarefied to an even depth of 5,861 sequences per sample. Data shown are the averages of three biological samples. A, Rarefaction curves depicting the mean number of OTUs in each group of samples as a function of the number of sequences. B, Box plots illustrating Shannon Index diversity in each group. C, PCoA plot calculated using Bray-Curtis dissimilarity matrix. ME3, P11, and P03 refer to the different sample sites. SW: sea water; NBW: near sea water; OSS: oxic surface sediment; T0: natural samples; T1: enriched samples in the incubation experiment; T1C: control in the incubation experiment.

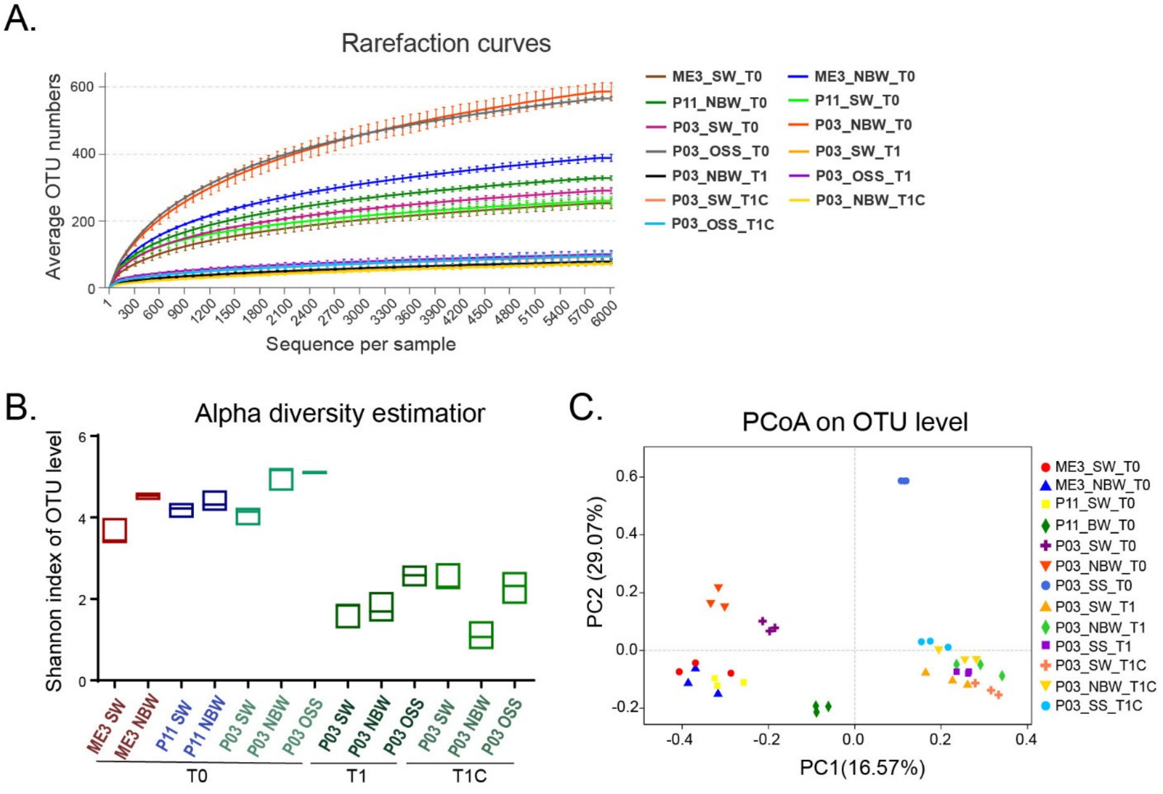

Fig. S2 Heatmap depicting the 50 most abundant genera from the 16S rRNA gene amplicon sequencing data from natural ECS samples (T0), the enriched (T1) and control incubation experiments after 14 days. *, $p < 0.05$ in Student's *t*-test comparing to the TIC samples.

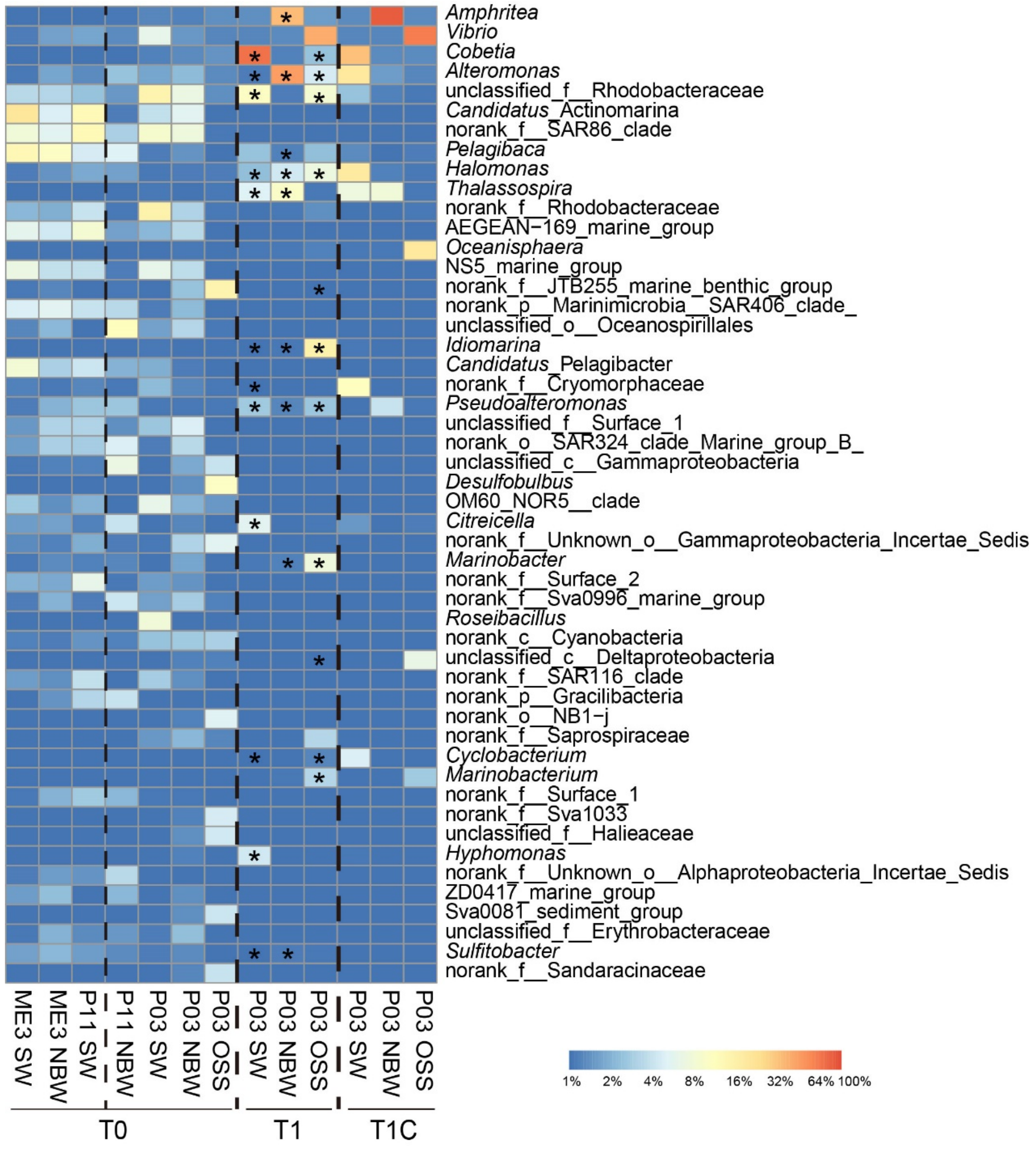

Fig. S3 Relative abundance for each significant taxon in the LEfSe analysis of T1 & T1C samples. Error bars represent standard deviation from the mean value.

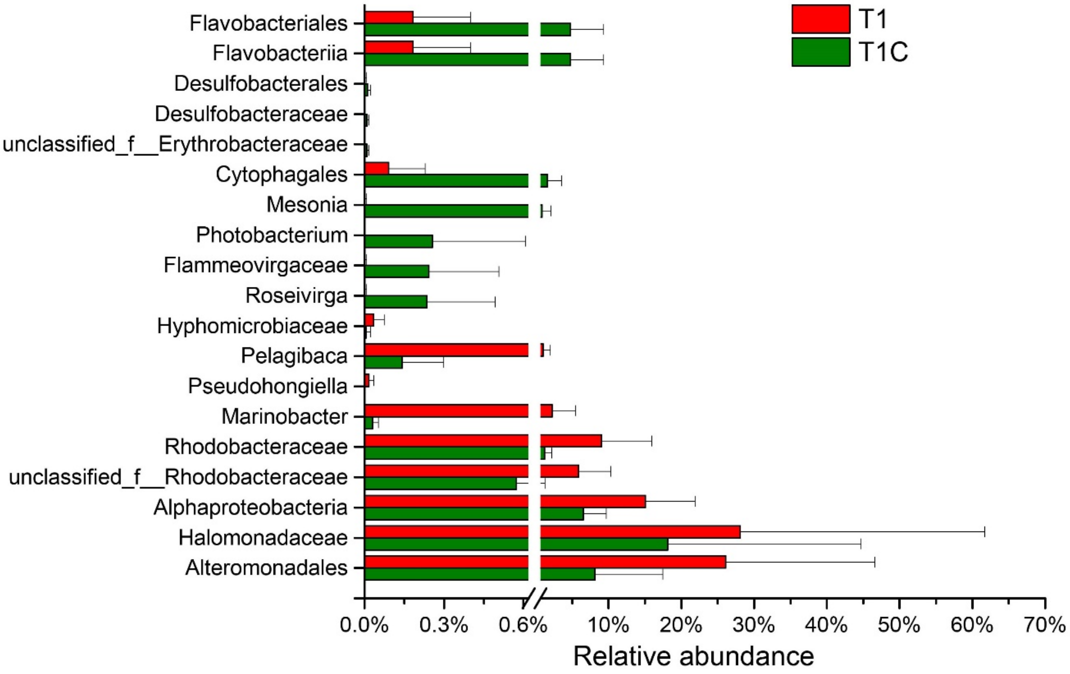

Fig. S4 Composition of eukaryotic plastid 16S rRNA genes in P03 SW and NBW samples. Total numbers of eukaryotic plastid 16S rRNA sequences identified in P03 SW and NBW were 339 ± 302 and 383 ± 230.

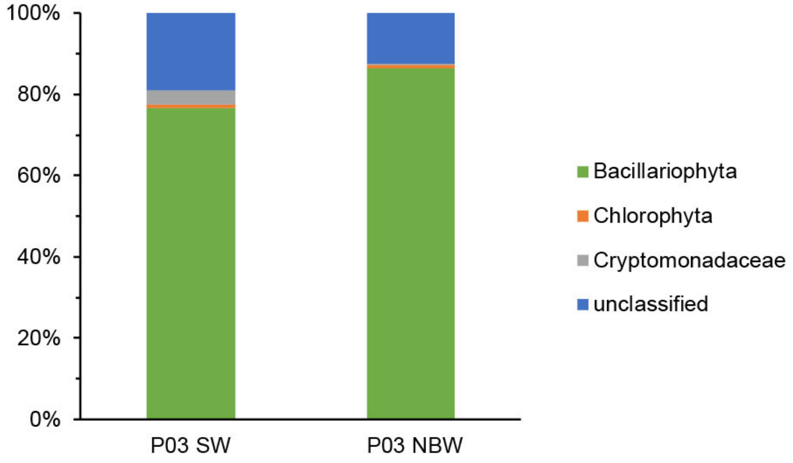

Fig. S5 Canonical correspondence analysis (CCA) on bacterial communities at family level in samples of incubation experiments.

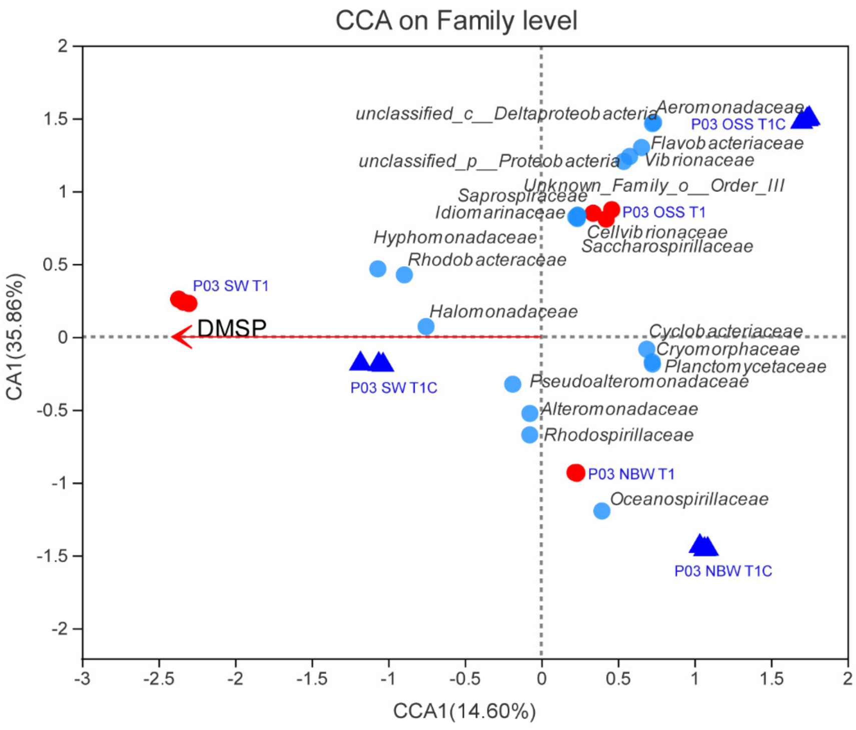

Fig. S6 Amino acid tree of representative DsyB and MmtN OTU sequences from clone libraries. Stars represent the source of different DsyB and MmtN OTUs. Non-functional DsyB and MmtN sequences were indicated in bold. Branch lengths are measured in the number of substitutions per site, as indicated on the scale bar.

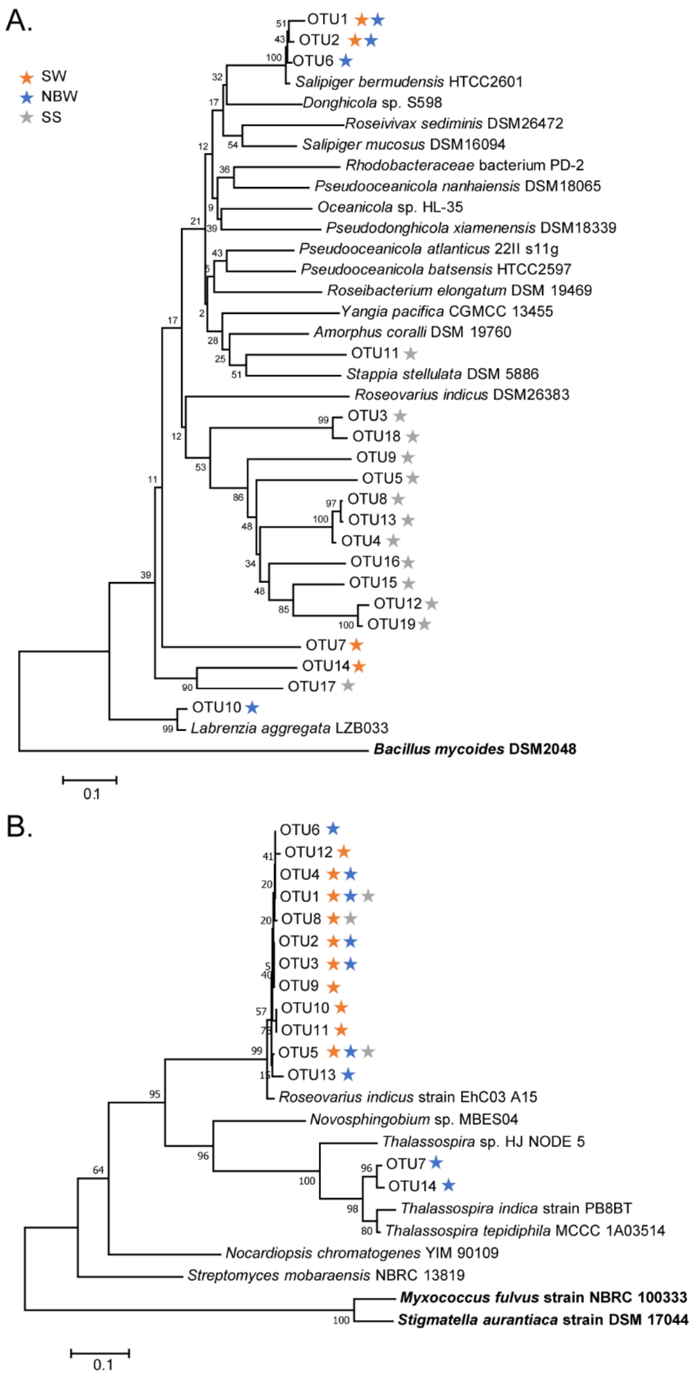

Reference

Alcolombri, U., Ben-Dor, S., Feldmesser, E., Levin, Y., Tawfik, D. S., and Vardi, A. (2015). Identification of the algal dimethyl sulfide-releasing enzyme: A missing link in the marine sulfur cycle. *Science.* doi:10.1126/science.aab1586.

Balch, W. E., Fox, G. E., Magrum, L. J., Woese, C. R., and Wolfe, R. S. (1979). Methanogens: reevaluation of a unique biological group. *Microbiol. Rev.*

Burkhardt, I., Lauterbach, L., Brock, N. L., and Dickschat, J. S. (2017). Chemical differentiation of three DMSP lyases from the marine: *Roseobacter* group. *Org. Biomol. Chem.* doi:10.1039/c7ob00913e.

Caporaso, J. G., Lauber, C. L., Walters, W. A., Berg-Lyons, D., Huntley, J., Fierer, N., et al. (2012). Ultra-high-throughput microbial community analysis on the Illumina HiSeq and MiSeq platforms. *ISME J.* doi:10.1038/ismej.2012.8.

Choi, D. H., Park, K. T., An, S. M., Lee, K., Cho, J. C., Lee, J. H., et al. (2015). Pyrosequencing revealed sar116 clade as dominant dddp-containing bacteria in oligotrophic nw pacific ocean. *PLoS One*. doi:10.1371/journal.pone.0116271.

Curson, A. R. J., Fowler, E. K., Dickens, S., Johnston, A. W. B., and Todd, J. D. (2012). Multiple DMSP lyases in the γ-proteobacterium *Oceanimonas doudoroffii*. *Biogeochemistry*. doi:10.1007/s10533-011-9663-2.

Curson, A. R. J., Liu, J., Bermejo Martínez, A., Green, R. T., Chan, Y., Carrión, O., et al. (2017). Dimethylsulfoniopropionate biosynthesis in marine bacteria and identification of the key gene in this process. *Nat. Microbiol.* 2, 17009. doi:10.1038/nmicrobiol.2017.9.

Curson, A. R. J., Rogers, R., Todd, J. D., Brearley, C. A., and Johnston, A. W. B. (2008). Molecular genetic analysis of a dimethylsulfoniopropionate lyase that liberates the climate-changing gas dimethylsulfide in several marine α-proteobacteria and *Rhodobacter sphaeroides*. *Environ. Microbiol.* doi:10.1111/j.1462-2920.2007.01499.x.

Curson, A. R. J., Sullivan, M. J., Todd, J. D., and Johnston, A. W. B. (2011a). DddY, a periplasmic dimethylsulfoniopropionate lyase found in taxonomically diverse species of *Proteobacteria*. *ISME J.* doi:10.1038/ismej.2010.203.

Curson, A. R. J., Todd, J. D., Sullivan, M. J., and Johnston, A. W. B. (2011b). Catabolism of dimethylsulphoniopropionate: Microorganisms, enzymes and genes. *Nat. Rev. Microbiol.* 9, 849–859. doi:10.1038/nrmicro2653.

Curson, A., Williams, B., Pinchbeck, B., Sims, L., Bermejo Martínez, A., Rivera, P., et al. (2018). DSYB catalyses the key step of dimethylsulfoniopropionate biosynthesis in many phytoplankton. *Nat. Microbiol.* doi:10.1038/s41564-018-0119-5.

De Souza, M. P., and Yoch, D. C. (1995). Comparative physiology of dimethyl sulfide production by dimethylsulfoniopropionate lyase in *Pseudomonas doudoroffii* and *Alcaligenes* sp. strain M3A. *Appl. Environ. Microbiol.*

DeLong, E. F. (1992). Archaea in coastal marine environments. *Proc. Natl. Acad. Sci.* 89, 5685–5689. doi:10.1073/pnas.89.12.5685.

Howard, E. C., Henriksen, J. R., Buchan, A., Reisch, C. R., Bürgmann, H., Welsh, R., et al. (2006). Bacterial taxa that limit sulfur flux from the ocean. *Science (80-.).* doi:10.1126/science.1130657.

Howard, E. C., Sun, S., Biers, E. J., and Moran, M. A. (2008). Abundant and diverse bacteria involved in DMSP degradation in marine surface waters. *Environ. Microbiol.* doi:10.1111/j.1462-2920.2008.01665.x.

Howard, E. C., Sun, S., Reisch, C. R., Del Valle, D. A., Bürgmann, H., Kiene, R. P., et al. (2011). Changes in dimethylsulfoniopropionate demethylase gene assemblages in response to an induced phytoplankton bloom. *Appl. Environ. Microbiol.* doi:10.1128/AEM.01457-10.

Kageyama, H., Tanaka, Y., Shibata, A., Waditee-Sirisattha, R., and Takabe, T. (2018). Dimethylsulfoniopropionate biosynthesis in a diatom *Thalassiosira pseudonana*: Identification of a gene encoding MTHB-methyltransferase. *Arch. Biochem. Biophys.* doi:10.1016/j.abb.2018.03.019.

Lane, D. J., Pace, B., Olsen, G. J., Stahl, D. A., Sogin, M. L., and Pace, N. R. (1985). Rapid determination of 16S ribosomal RNA sequences for phylogenetic analyses. *Proc. Natl. Acad. Sci. U. S. A.* 82, 6955–9. doi:10.1073/PNAS.82.20.6955.

Lei, L., Cherukuri, K. P., Alcolombri, U., Meltzer, D., and Tawfik, D. S. (2018). The Dimethylsulfoniopropionate (DMSP) lyase and lyase-like cupin family consists of bona fide DMSP lyases as well as other enzymes with unknown function. *Biochemistry*. doi:10.1021/acs.biochem.8b00097.

Li, C. Y., Chen, X. L., Xie, B. Bin, Su, H. N., Qin, Q. L., and Zhang, Y. Z. (2014). Reply to Tawfik et al.: DddQ is a dimethylsulfoniopropionate lyase involved in dimethylsulfoniopropionate catabolism in marine bacterial cells. *Proc. Natl. Acad. Sci. U. S. A.* doi:10.1073/pnas.1403460111.

Li, C. Y., Zhang, D., Chen, X. L., Wang, P., Shi, W. L., Li, P. Y., et al. (2017). Mechanistic insights into dimethylsulfoniopropionate lyase DddY, a new member of the cupin superfamily. *J. Mol. Biol.* doi:10.1016/j.jmb.2017.10.022.

Sun, J., Todd, J. D., Thrash, J. C., Qian, Y., Qian, M. C., Temperton, B., et al. (2016). The abundant marine bacterium *Pelagibacter* simultaneously catabolizes dimethylsulfoniopropionate to the gases dimethyl sulfide and methanethiol. *Nat. Microbiol.* doi:10.1038/nmicrobiol.2016.65.

Todd, J. D., Curson, A. R. J., Dupont, C. L., Nicholson, P., and Johnston, A. W. B. (2009). The dddP gene, encoding a novel enzyme that converts dimethylsulfoniopropionate into dimethyl sulfide, is widespread in ocean metagenomes and marine bacteria and also occurs in some Ascomycete fungi. *Environ. Microbiol.* doi:10.1111/j.1462-2920.2009.01864.x.

Todd, J. D., Curson, A. R. J., Kirkwood, M., Sullivan, M. J., Green, R. T., and Johnston, A. W. B. (2011). DddQ, a novel, cupin-containing, dimethylsulfoniopropionate lyase in marine roseobacters and in uncultured marine bacteria. *Environ. Microbiol.* doi:10.1111/j.1462-2920.2010.02348.x.

Todd, J. D., Curson, A. R. J., Nikolaidou-Katsaraidou, N., Brearley, C. A., Watmough, N. J., Chan, Y., et al. (2010). Molecular dissection of bacterial acrylate catabolism - unexpected links with dimethylsulfoniopropionate catabolism and dimethyl sulfide production. *Environ. Microbiol.* doi:10.1111/j.1462-2920.2009.02071.x.

Todd, J. D., Kirkwood, M., Newton-Payne, S., and Johnston, A. W. B. (2012). DddW, a third DMSP lyase in a model *Roseobacter* marine bacterium, Ruegeria pomeroyi DSS-3. *ISME J.* doi:10.1038/ismej.2011.79.

Todd, J. D., Rogers, R., You, G. L., Wexler, M., Bond, P. L., Sun, L., et al. (2007). Structural and regulatory genes required to make the gas dimethyl sulfide in bacteria. *Science (80-.).* doi:10.1126/science.1135370.

Van Der Maarel, M. J. E. C., Van Bergeijk, S., Van Werkhoven, A. F., Laverman, A. M., Meijer, W. G., Stam, W. T., et al. (1996). Cleavage of dimethylsulfoniopropionate and reduction of acrylate

by *Desulfovibrio acrylicus* sp. nov. *Arch. Microbiol.* doi:10.1007/s002030050363.

Wang, P., Chen, X. L., Li, C. Y., Gao, X., Zhu, D. Y., Xie, B. Bin, et al. (2015). Structural and molecular basis for the novel catalytic mechanism and evolution of DddP, an abundant peptidase-like bacterial Dimethylsulfoniopropionate lyase: A new enzyme from an old fold. *Mol. Microbiol.* doi:10.1111/mmi.13119.

Yin, Q., Fu, B., Li, B., Shi, X., Inagaki, F., and Zhang, X. H. (2013). Spatial variations in microbial community composition in surface seawater from the ultra-oligotrophic center to rim of the South Pacific Gyre. *PLoS One* 8. doi:10.1371/journal.pone.0055148.
